# Landscape factors influencing honey bee colony behavior in Southern California commercial apiaries

**DOI:** 10.1038/s41598-020-61716-6

**Published:** 2020-03-19

**Authors:** William G. Meikle, Milagra Weiss, Eli Beren

**Affiliations:** Carl Hayden Bee Research Center, USDA-ARS Tucson, AZ USA

**Keywords:** Agroecology, Behavioural ecology

## Abstract

Colony brood levels, frames of bees (adult bee mass) and internal hive temperature were monitored for 60 colonies for each of two years as they were moved from agricultural, tree crop and mountain landscapes in southern California to blueberry and almond pollination sites. Hive weight was also continuously monitored for 20 of those hives for 6 weeks for both years, during commercial pollination. Pesticide residues in wax, honey and beebread samples were analyzed by composite apiary samples. While colonies in mountain sites had more adult bees and brood than those in agricultural sites in August, by October brood levels were higher in colonies from agricultural sites. Though hives from different original landscapes differed in size in October, hive assessments revealed no differences between the groups after co-wintering when graded for commercial almond pollination. Beebread from hives in agricultural sites had greater agrochemical diversity and in general higher pesticide hazard quotients than those from mountain sites, but those hives also had higher and more constant temperatures from September until January than hives from mountain sites. Hives placed in commercial almond pollination gained on average 287 g per d, compared to an average loss of 68 g per d for colonies in commercial blueberry pollination, although weight data indicated greater foraging effort by colonies in blueberries, possibly due to the proximity and abundance of almond pollen during bloom. Temperature monitoring was effective at distinguishing hive groups and had the best overall value in terms of equipment, installation, colony disturbance and information yield.

## Introduction

Honey bee colonies are highly adaptable to new environments, owing to their organization and behavioral plasticity. Colonies change their energy and nutrient investments in brood production, foraging activity, thermoregulation and other colony-level behaviors in response to changes in external conditions, such as weather and forage availability^1^, Commercial beekeeping operations take advantage of this adaptability by moving colonies among different sites for honey production or pollination contracts during the course of the year, sometimes considerable distances and to other environments^2^. These new environments may differ with respect to ambient weather, available forage, agrochemical exposure, and colony density, which in turn can affect robbing, disease and pest pressures.

Commercial pollination contracts are typically established for the pollination of seed crops such as alfalfa and fruit crops such as blueberries, as well as almonds^3^. While these crops, which are typically irrigated, can provide much needed forage when local uncultivated plants provide little or none, they are also subject to frequent treatments with pesticides, including insecticides, fungicides and herbicides^4^. Commercial honey bee colonies in general are frequently exposed to pesticides^5,6^, and exposure to many classes of agrochemicals has been found to affect bee colony health and behavior^4,7–10^. The exposure can occur in several ways, such as via collected pollen and nectar, or via physical exposure and subsequent contamination of beeswax, a lipophilic material, in the hive^10^. Honey is the main source of carbohydrates consumed by all classes of workers while beebread is consumed predominantly by young workers, or “nurse” bees, which provision brood and provide nutrients to other bees in the colony^11^.

There are many methods for measuring impact of an environmental factor on a colony. Commercial beekeepers often use a rapid visual assessment, for example, whereas researchers may use more precise (and more time-consuming) methods or sensor-based methods. Sensor-based measurements can be powerful tools because of the large amounts of longitudinal data generated, and they can also provide information on the timing of particular events, such as a weather event or the death of a colony^12^. Weight and temperature sensors have been used in longitudinal field studies of commercial colonies to detect differences in colony growth and behavior among different landscapes^13–15^. In this study we incorporated, to some degree, all those approaches. Colonies were rapidly assessed for frames of bees, brood surface area estimated using image analysis of frame photographs, and hives were monitored using temperature sensors and hive scales. Internal hive temperature, measured using small, unobtrusive self-contained sensors, has been found to be highly informative as a measure of colony health and activity. Honey bee colonies tend to maintain stable temperatures (34–35 °C) in and around the brood nest^16,17^ and while temperatures tend to be more variable in the absence of brood, at no point do bee colonies cease thermoregulation entirely. Colony thermoregulation is affected by within-colony genetic diversity^18^, subspecies^19^, phenological status^20^ and pesticide exposure^8,21^, in addition to simply colony size^15^. Finally, although the installation of hive scales is not feasible in all situations, continuous weight data has been shown to be rich in information on colony behavior and growth^8,16,22^.

**Figure 1:**
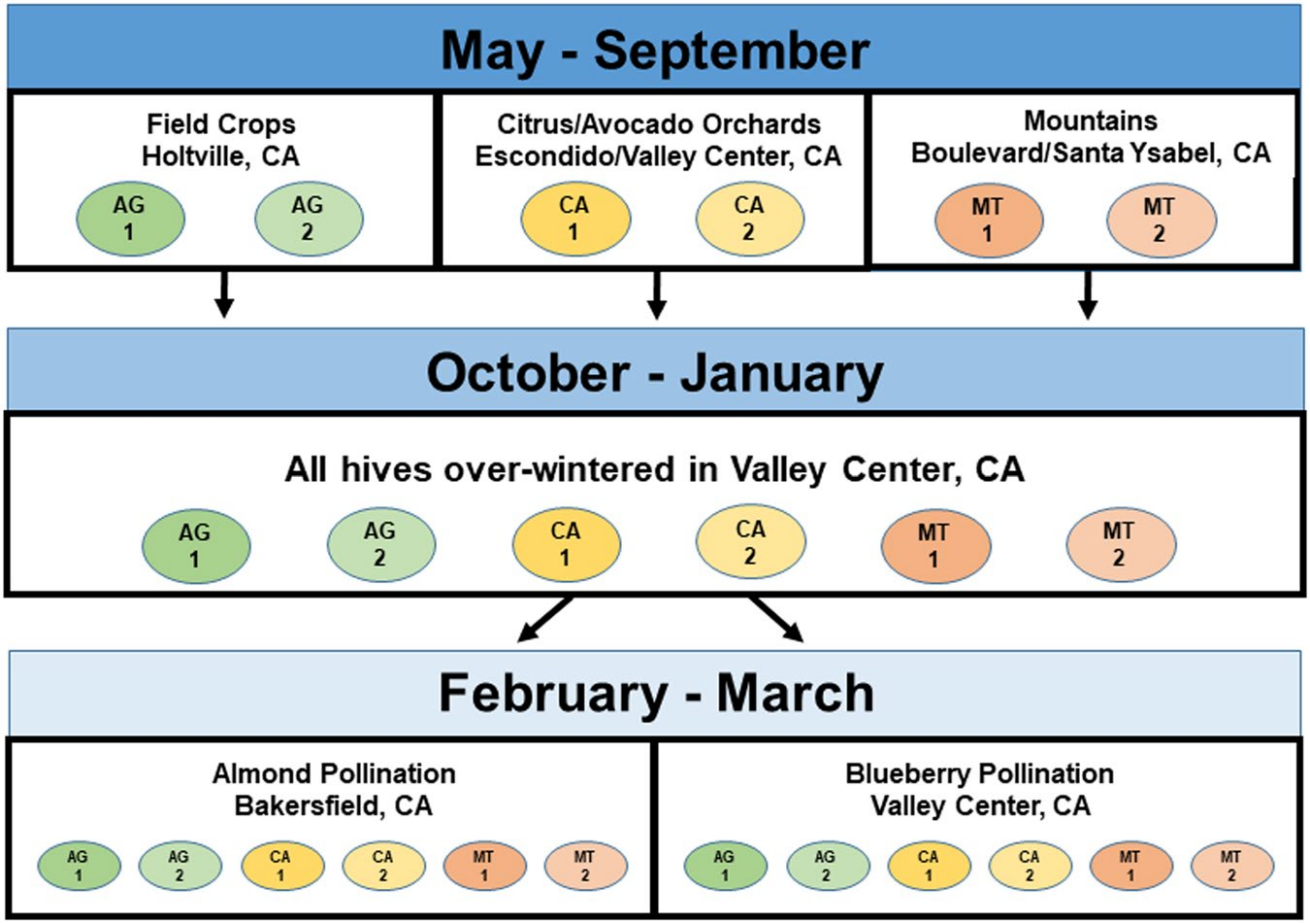
Schematic diagram of the experimental design for both years of the study.

The objectives of this study were: (1) to assess and monitor the growth and activity of commercial bee colonies subjected to different landscapes, using both hive assessment and sensor-based methods; and (2) to explore within-hive factors that may help explain any observed differences. For the purposes of this study we consider “landscape” to be areas with roughly similar temperature, altitude and vegetation profiles within the expected foraging range of an apiary (the mean foraging distance of bee colonies has been shown to vary from 1.0 to 5.5 km^23^). Commercial bee colonies were identified in one of three landscape environments in Southern California: sites in field crop agriculture in the Imperial Valley (AG1 and AG2 apiaries in 2016 and AG3 and AG4 apiaries in 2017); sites with significant acreage of citrus and avocado orchards (CA1 and CA2 apiaries for 2016 and CA3 and CA4 apiaries for 2017); and sites at higher-altitude with essentially no agriculture of any kind (MT1 and MT2 apiaries for 2016 and MT3 and MT4 apiaries for 2017). After several months in those landscapes, hereafter “original landscapes,” all colonies were moved to the same site in a blueberry farm for several months, where monitoring continued. By monitoring hives kept at different sites during their period of growth and resource acquisition, i.e. late spring and summer, and then moving them all to a single (new) site, it may be possible to observe whether and how the original landscape exposure can influence colony health and activity. Low- and mid-altitude localities in the southern half of the US, such as southern California through southern Texas, seldom have the extended cold periods and shortened photoperiods of those in the northern half^24^, and so honey bee colonies in the south tend to be actively foraging for all but a month or two of the year. Finally, hives were divided into two groups (independent of their size) with one group moved to almond pollination and the other group remaining at the common site (Fig. 1), and their growth and activity monitored in the new situations. The study thus comprised three main phases: from August to September, or the “Original landscape” phase when the monitoring of colonies was initiated; from October to January or the “Pre pollination” phase; and from February to March or the “Pollination” phase. By observing bee colony phenology and behavior in different landscapes where we also measured agrochemical and pest stressors, we hoped to gain some insight into the relative impacts of these stressors in a commercial context in Southern California.

## Results

Land use categories are shown for all locations (Table 1). Crop forage in the Imperial Valley from June to September included alfalfa (*Medicago sativa*), sugar beets (*Beta vulgaris*), and other forage plants included tamarisk (*Tamarix* spp.), and grasses such as Bermuda (*Cynodon dactylon*) and Sudan (*Sorghum x drummondii*). Forage in suburban Escondido during that period was likely limited to grasses, and wild and managed plants, since the main forage sources, citrus, avocado and eucalyptus, flower in the winter and spring. The “mountain” sites had no significant commercial agriculture with foraging distance; main forage plants, observed by the team were California buckwheat (*Eriogonum fasciculatum*), mountain sage (*Salvia regla*), and broomweed (*Gutierrezia sarothrae*) (D. Winter pers. comm.). MT apiaries the second year were slightly higher (1100 m) than those the previous year (800–900 m) and forage included redshank (*Adenostoma sparsifolium*) as well as buckwheat, mountain sage and broomweed (D. Winter pers. comm.).

**Table 1:** Average percentage (±standard error) for a circle with a radius of approximately 1.7 km (=approximately 910 ha) of land around the apiaries in this study (n = 4 for AG, CA and MT; n = 1 for blueberries and almonds), according to the Cropscape web site.

Type	Category	Landscape				
		AG	CA	MT	Blue	Alm
Crop	Alfalfa	26.0 ± 8.2	<0.1	—	0.2	<0.1
Crop	Blueberries	—	—	—	—	0.2
Crop	Broccoli	2.5 ± 1.1	—	—	—	—
Crop	Cabbage	0.1 ± 0.1	—	—	—	—
Crop	Cantaloupes	<0.1	—	—	—	—
Crop	Carrots	2.9 ± 1.0	—	—	—	<0.1
Crop	Cauliflower	0.6 ± 0.5	—	—	—	—
Crop	Celery	<0.1	—	—	—	—
Crop	Corn	1.9 ± 1.6	—	—	—	0.1
Crop	Cotton	<0.1	—	—	—	<0.1
Crop	WinterWheat/Corn	—	—	—	—	<0.1
Crop	Garlic	—	—	—	—	0.5
Crop	Grapes	—	—	—	<0.1	11.6
Crop	Greens	10.4 ± 5.1	—	—	—	—
Crop	Herbs	0.1 ± 0.0	—	—	—	—
Crop	Lettuce	6.2 ± 3.4	—	—	—	—
Crop	Onions	6.9 ± 3.1	—	—	—	—
Crop	Other Hay/Non Alfalfa	3.8 ± 2.0	<0.1	<0.1	<0.1	<0.1
Crop	Potatoes	<0.1	—	—	—	—
Crop	Sod/Grass Seed	0.1 ± 0.0	—	—	—	—
Crop	Sugarbeets	12.0 ± 5.5	—	—	—	—
Crop	Sugarcane	0.1	—	—	—	—
Crop	Sunflowers	0.9 ± 0.5	—	—	—	—
Crop	Triticale	<0.1	—	—	—	—
Crop	Watermelons	0.1	—	—	—	—
Crop	Winter Wheat	3.3 ± 1.6	—	—	<0.1	<0.1
Other	Barren	1.1 ± 1.0	0.1 ± 0.1	0.5 ± 0.2	<0.1	<0.1
Other	Dev./High Intens.	<0.1	0.1 ± 0.0	—	—	1.3
Other	Dev./Low Intens.	4.2 ± 0.4	5.8 ± 3.1	0.3 ± 0.3	1.0	1.6
Other	Dev./Medium Intens.	0.2 ± 0.1	2.1 ± 1.0	0.2	—	1.9
Other	Dev./Open Space	1.6 ± 0.4	16.4 ± 5.2	8.2 ± 1.5	4.2	3.8
Other	Evergreen Forest	<0.1	0.5 ± 0.2	0.1 ± 0.1	1.0	—
Other	Fallow/Idle Cropland	0.4 ± 0.2	—	—	—	5.5
Other	Grass/Pasture	<0.1	9.9 ± 3.5	25.9 ± 4.6	9.7	0.3
Other	Herbaceous Wetlands	0.2 ± 0.0	0.1 ± 0.0	0.4 ± 0.2	<0.1	—
Other	Mixed Forest	—	0.5 ± 0.2	0.6 ± 0.3	0.2	—
Other	Open Water	<0.1	0.2 ± 0.0	0.1 ± 0.0	0.1	<0.1
Other	Shrubland	13.8 ± 9.3	41.1 ± 9.5	63.9 ± 4.3	46.5	1.0
Other	Woody Wetlands	1.5 ± 0.5	0.5 ± 0.1	0.1 ± 0.0	0.1	<0.1
tree	Almonds	—	—	—	—	71.5
tree	Avocados	—	12.7 ± 3.4	—	26.5	—
tree	Citrus	—	9.6 ± 4.9	—	11.1	—
tree	Oranges	—	0.7 ± 0.3	—	<0.1	0.1
tree	Other Tree Crops	0.3 ± 0.1	0.1 ± 0.0	0.1 ± 0.0	0.1	1.2
tree	Pomegranates	—	—	—	—	<0.1

“Blue” = blueberry farm, “Alm” = almond orchard, “Dev.” = developed, “intens.” = intensity. Values without standard error indicate that only one sample had the reported use category.

In summary:

1. AG sites were about 78 ± 11% field crops, <1% tree crops and 22 ± 11% other kinds of land use;

2. CA sites were about 6 ± 0% field crops, 23 ± 2% tree crops and 77 ± 2% other kinds of land use; and

3. MT sites were <1% field or tree crops and almost entirely other kinds of land use.

Some caveats are in order with respect to the analysis using Cropscape data, at least in the case of the “blueberry farm” near Valley Center. Cropscape did not indicate any blueberries at that site, although blueberry production is a layer on the Cropscape map, but numerous visits by the research team to that area indicated several ha of blueberries.

Response variables with respect to bee hives in this study included frames of bees (FOB), brood surface area (cm^2^), Varroa density (mites per 100 bees), hive temperature, divided into average temperature and temperature variability. In addition, continuous weight data were gathered on 40 hives, and that data used to estimate average daily weight change, nightly hive weight change, the start (dawn) and stop (dusk) of the daily colony activity cycle, and foraging effort. Apiary-level data included land use estimates and agrochemical diversity and concentrations in hive products (wax, honey and bee bread). All raw data on land use categories, hive assessment, temperature and weight are available in the Supplementary File.

### Hive assessment

Brood surface area changed across the three time points (August, October and March) (Table 2 and Supplementary Table S1). The three original landscapes, Imperial Valley agriculture sites (AG), sites with comparatively large acreage of citrus and avocado orchards (CA) and non-agriculture higher altitude sites (MT) were significantly different in August, at the start of both experiments. The AG colonies had significantly less brood surface area than either the CA or MT colonies. In October brood surface areas were again significantly different, but the AG colonies had significantly more brood than the MT colonies. Both August and October brood production were also significantly different with respect to Year. On the last sample date in March, after almond pollination, no significant differences were observed among the original landscapes.

**Table 2:** Post hoc contrast results for MANOVA analyses of brood surface area (±s.e.), frames of bees (±s.e.) and temperature averages and amplitudes (±s.e) by original landscape for each sampling occasion across the two years prior to pollination.

Original landscape	Brood surface area (cm^2^)			
	August		October	
Agriculture	1503 ± 155 a		936 ± 13 a	
Citrus Avocado	2086 ± 123 b		969 ± 24 a	
Mountain	1963 ± 446 b		539 ± 243 b	
	**Frames of adult bees (FOB)**			
	**August**		**October**	**January**
Agriculture	12.4 ± 0.6 a		10.0 ± 0.2 a	8.8 ± 1.4 a
Citrus Avocado	12.9 ± 0.4 ab		9.0 ± 1.0 a	7.1 ± 1.7 a
Mountain	13.8 ± 1.2 b		10.8 ± 0.9 a	7.9 ± 0.5 a
	**Avg. daily temperature (°C)**		**Temperature amplitude (°C)**	
	**Aug.-Oct**.	**Nov.-Jan**.	**Aug.-Oct**.	**Nov.-Jan**
Agriculture	35.1 ± 0.04 a	32.4 ± 0.80 a	0.3 ± 0.03 a	0.7 ± 0.14 a
Citrus Avocado	34.5 ± 0.03 a	28.5 ± 1.50 b	0.6 ± 0.01 b	2.2 ± 0.23 b
Mountain	34.2 ± 0.09 a	28.2 ± 0.30 ab	0.4 ± 0.03 b	1.4 ± 0.17 b

Treatment groups within response variable and time period with no letters in common are significantly different at α = 0.05.

Comparisons of frames of bees (FOB) showed differences among original landscape groups, but only for the August inspection (Table 1 and Supplementary Table S2). As with brood surface area, colonies in the MT group had significantly more bees than did colonies in the AG group. FOB data could be gathered without serious hive disturbance, so data were gathered in January as well, and the October to January data were included in the same analysis. Neither original landscape nor year were significant in the October-January analysis. No fixed factors were significant after almond pollination.

### Hive temperature

**Figure 2:**
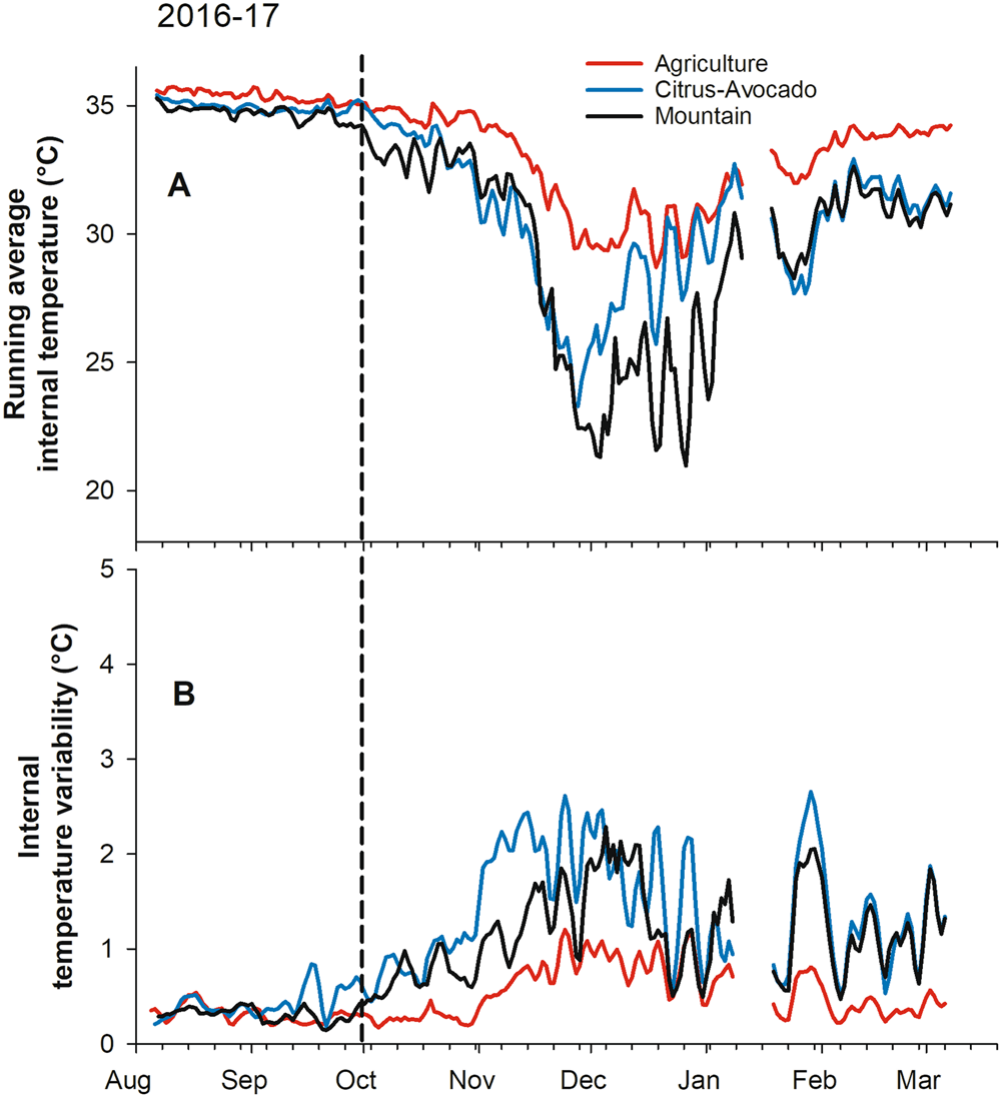
Internal hive temperature data (°C) for commercial beehives in three landscapes (agriculture, citrus-avocado and mountain) in Southern California from August 2016 to March 2017. (**A**) Running average temperature; (**B**) Internal temperature variability. The vertical dashed line shows the approximate date hives were moved from the original landscapes to the common blueberry site (near Valley Center, CA).

**Figure 3:**
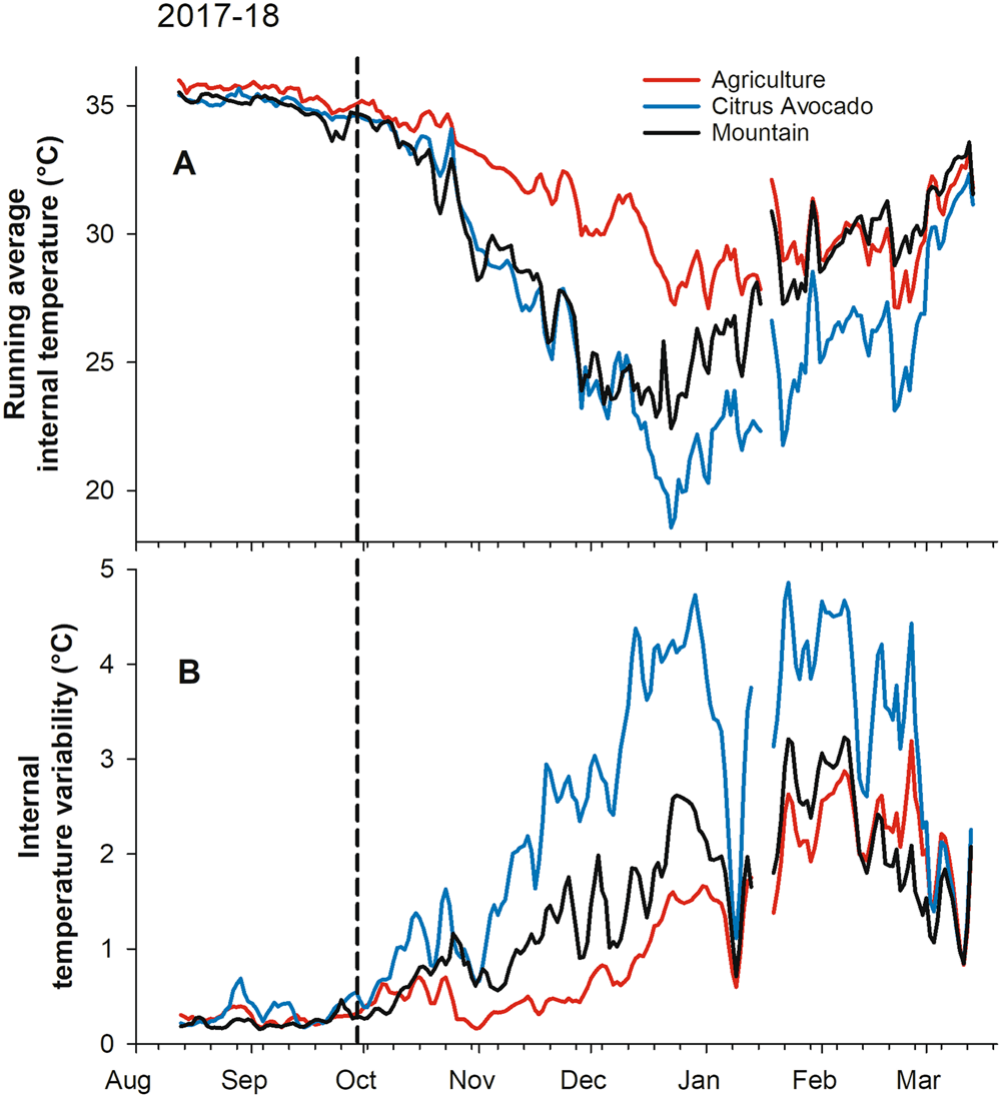
Internal hive temperature data (°C) for commercial beehives in three landscapes (agriculture, citrus-avocado and mountain) in Southern California from August 2017 to March 2018. (**A**) Running average temperature; (**B**) Internal temperature variability. The vertical dashed line shows the approximate date hives were moved from the original landscapes to the common blueberry site (near Valley Center, CA).

Within-day temperature amplitudes from the beginning of the experiment in mid-August until the end of October were significantly affected by original landscape across both years of the study but average daily temperature was not (Figs. 2 and 3, Supplementary Table S3). Post hoc contrasts showed that bee colonies kept in the Imperial Valley during the summer had lower temperature amplitudes during that period than colonies placed in the CA environment (Table 1). Restricting the analysis to November to mid-January, when the challenge to maintain temperatures within the hive is greatest, the AG colonies maintained average temperatures 3.9 to 4.2 °C higher, and temperature variability 0.7–1.4 °C lower, than colonies at either of the other sites over both years of the study. Considering the entire period from mid-August to mid-January and using the “slices” function in SAS, significant treatment effects were observed with respect to average hive temperature for 74 of the next 80 days after 29 October, but not before that date. Significant treatment effects were observed with respect to temperature amplitude for 112 of 115 days after 16 September.

**Figure 4:**
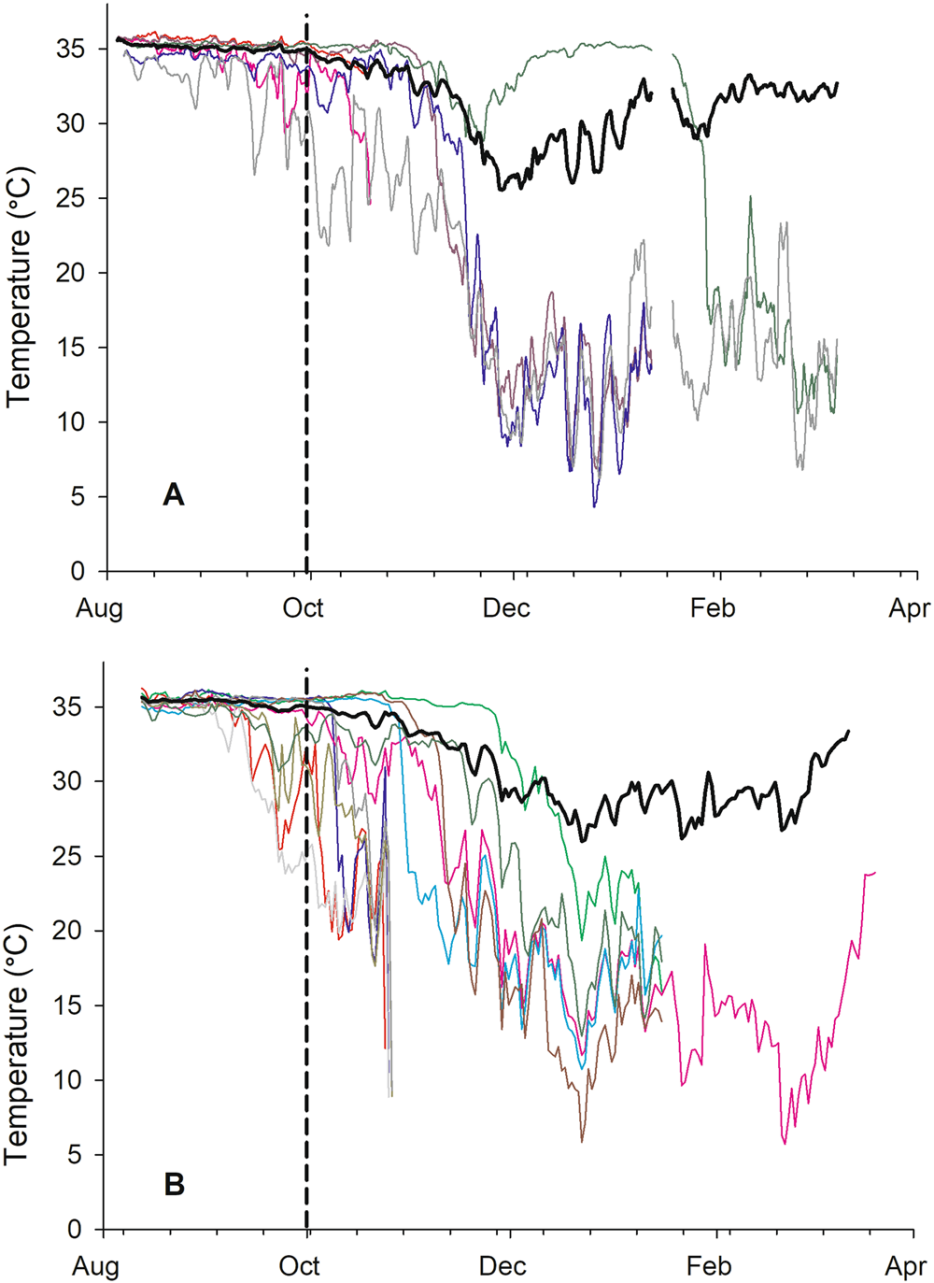
Hourly temperatures for dying hives during the course of the experiments. (**A**) August 2016- March 2017; (**B**) August 2017- March 2018. Solid line shows average temperature of all remaining hives after data for the dying hives had been entirely removed. The vertical dashed line shows the approximate date hives were moved from the original landscapes to the common blueberry site (near Valley Center, CA).

Neither temperature average nor amplitude were significantly different among original landscape groups when analyses were restricted from mid-January to the end of almond pollination in early March, although temperature averages were different between pollination sites (Supplementary Table S4). However, those temperature differences were inconsistent between the two years with temperature averages higher in almonds in 2017 but lower in 2018 (Table 3). Colonies that failed during the course of the study generally failed in late fall and early winter, and exhibited distinctive temperature patterns when compared to overall colony averages without those colonies (Fig. 4). Colonies were removed from the study once their death had been confirmed by visual assessment.

**Table 3:** Average brood surface area (cm^2^) and adult bee population (frames of bees) observed in March of each year, and temperature averages and amplitudes (±s.e) observed from mid-January to March for each year, for hives placed in almond or blueberry pollination.

Year	Orig. landsc.	No. colonies		Brood area		FOB	
		Alm	Blu	Almonds	Blueberries	Almonds	Blueberries
2017	AG	9	7	3303 ± 545	3398 ± 469	18.4 ± 1.6	16.0 ± 2.0
	CA	10	10	3833 ± 274	2366 ± 555	13.0 ± 1.1	11.9 ± 2.0
	MT	9	10	3846 ± 417	3004 ± 573	10.7 ± 1.8	11.3 ± 1.0
2018	AG	8	10	2140 ± 566	2341 ± 502	10.1 ± 1.5	10.4 ± 1.2
	CA	8	8	2827 ± 318	3442 ± 462	11.3 ± 1.3	12.0 ± 1.6
	MT	8	9	3106 ± 263	1582 ± 294	13.5 ± 0.6	10.4 ± 1.1
				**Avg. daily temp. (°C)**		**Temp. ampl. (°C)**	
				**Almonds**	**Blueberries**	**Almonds**	**Blueberries**
2017	AG	9	7	35.2 ± 0.1	31.8 ± 2.1	0.2 ± 0.0	0.6 ± 0.3
	CA	10	10	34.8 ± 0.2	31.6 ± 2.1	0.2 ± 0.0	0.9 ± 0.4
	MT	9	10	35.1 ± 0.2	33.1 ± 1.4	0.2 ± 0.0	0.6 ± 0.4
2018	AG	8	10	28.7 ± 2.2	31.1 ± 1.5	3.2 ± 0.9	1.4 ± 0.5
	CA	8	8	27.7 ± 2.8	27.7 ± 2.5	2.7 ± 0.8	3.8 ± 1.2
	MT	8	9	28.7 ± 2.0	30.8 ± 2.0	2.9 ± 0.8	1.8 ± 0.7

Orig. landsc. = original landscape (see text for details); FOB = frames of adult bees; AG = agricultural sites; CA = citrus-almond sites; MT = mountain sites; Alm = almond site; Blu = blueberry site; Avg. daily temp. = average daily temperature; Temp. ampl. = daily temperature amplitude.

### Hive weight

**Figure 5:**
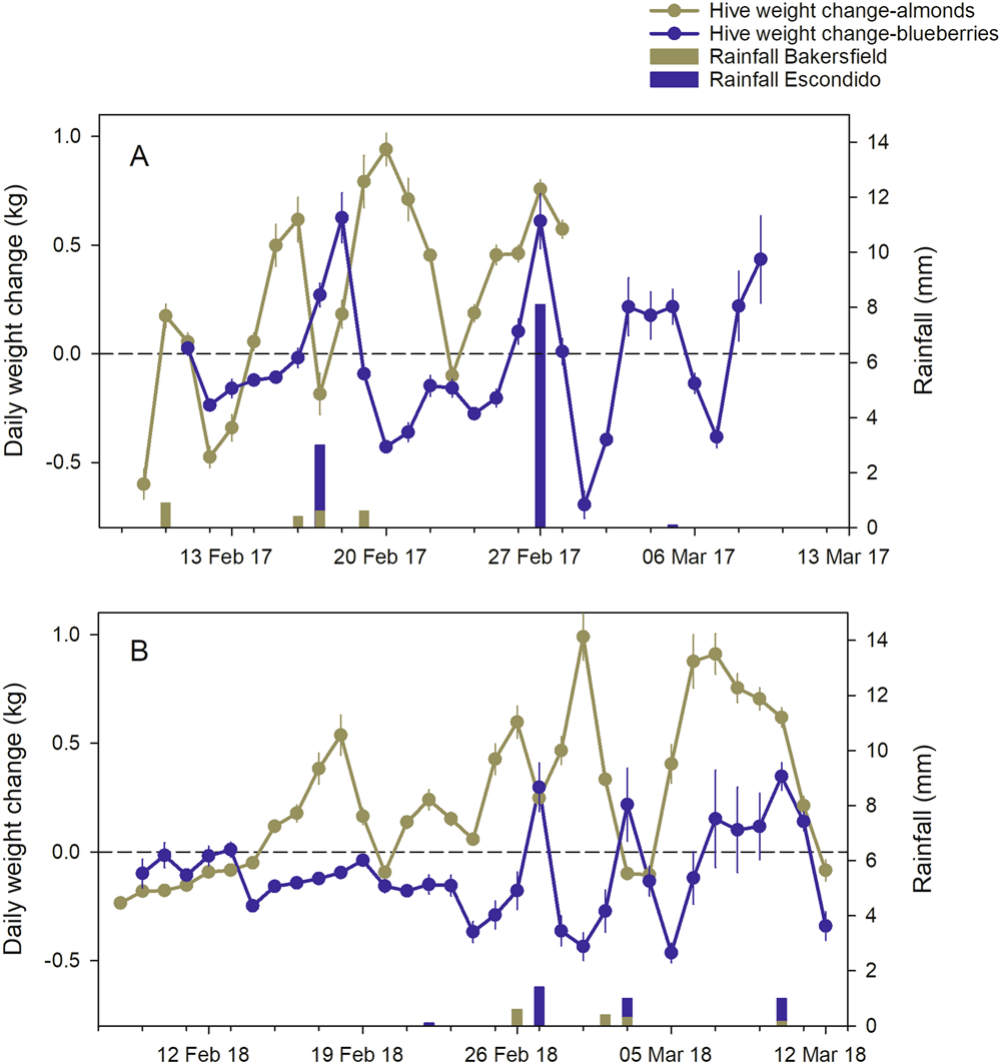
Average (±s.e.) between-day weight changes for hives placed in an almond orchard during pollination, and hives placed in a blueberry farm during that same period. Rainfall (bar graphs) is shown for comparison with weight changes. (**A**) February to March 2017; (**B**) February to March 2018.

Daily hive weight changes were significantly different between colonies sent to almond pollination and those retained in blueberry pollination (Table 4, Fig. 5, Supplementary Tables S5 and S6). Colonies placed in almond pollination gained on average 287 g per d between the two years of the study, and those retained in blueberry pollination lost on average 73 g per d. Distinct patterns in increasing and decreasing rates of hive weight change were compared to rainfall data for each location. Rainfall near Escondido was higher and of the seven peaks in hive weight gain across both years, five were associated with the five highest local rainfall events, suggesting that without rainfall, hive weight loss would have been higher. Of nine peaks on hive weight gain in almonds between the two years, only four were associated with rainfall and in almost all cases only partially. The hive weight change patterns in almonds may have been be related to factors such as the variety or local environment of the plants upon which the bees were foraging.

**Table 4:** Daily hive weight change, dawn and dusk parameters and estimated initial forager mass for hive weight data collected in 2017 and 2018.

Location	Year	No. hives	No. days	Daily hive weight change (g)	Piecewise regression			
					Dawn	Dusk	Night weight loss (g/h)	Forager mass (g)
Almond pollination	2017	10	20	315 ± 34	8:57 ± 0:06	17:14 ± 0:04	13.0 ± 1.4	244 ± 54
	2018	10	33	262 ± 21	8:56 ± 0:11	18:09 ± 0:03	12.2 ± 2.3	126 ± 37
Blueberry pollination	2017	10	29	−44 ± 25	8:24 ± 0:04	17:07 ± 0:08	9.6 ± 1.9	346 ± 57
	2018	9	32	−101 ± 19	8:36 ± 0:07	17:21 ± 0:07	3.7 ± 1.9	319 ± 26

Hives in almond pollination were placed near Bakersfield, CA, while hives in blueberry pollination were placed near Escondido, CA. Time shown is 24 h clock. Days with rainfall >3 mm are excluded. N = 10 hives per treatment group for each year. Values shown are ±s.e.

Dawn (t_D_), dusk, the night slope (S_N_) and the hive weight change due to initial forager departure (ΔF) were significantly different between pollination environments. Dawn break points were roughly consistent between the two years, with the colonies in blueberries beginning activity 20–33 minutes before those in almonds, and no differences were detected between years. Dusk break points were also different, with colonies in blueberries ceasing foraging 7–48 minutes earlier than those in almonds, but dusk times were also significantly different between years.

Night slopes were significantly different between colonies placed in almonds and those placed in blueberries. Hives placed in almonds lost, on average, about 12.6 g/h during the night and those in blueberries lost about 6.7 g/h. These differences likely reflect the quantity of nectar and pollen in the hives; the higher value for hives in almonds simply reflects the larger surface area of drying nectar and pollen than those in blueberries, which was reflected in the higher average daily hive weight change for hives in almonds discussed above. The initial forager mass, after subtracting an estimated value for the moisture loss during that period, were significantly lower for colonies placed in almonds compared to those placed in blueberries, after controlling for adult bee mass.

### Mite density

Mite levels were not significantly different among the original landscapes but they were significantly different between years (Supplementary Table S7). Mites were lower in the 2016–17 trial (0.33 ± 0.19 and 0.34 ± 0.24 mites per 100 bees for the August and October samples, respectively) than in the 2017–18 trial (1.94 ± 0.27 and 1.87 ± 0.32 mites per 100 bees for the August and October samples, respectively). After almond pollination in 2017 no mites were detected whereas in 2018 colonies placed in almonds had 0.21 ± 0.08 mites per 100 bees and those in blueberries had 0.73 ± 0.38 mites per 100 bees.

### Agrochemical residues

Three matrices were evaluated for residues: honey (or nectar), wax and beebread. A total of 68 compounds were detected, including fungicides, herbicides, insecticides, miticides, two synergist compounds and an insect repellent (Supplementary Table S8). Honey samples were evaluated over four sampling occasions in the 2016–17 experiment (Supplementary Table S9). DMPF (a breakdown product of amitraz, a miticide) was detected in all samples at concentrations ranging from 6 to 155 ppb. Flonicamid, a pyridine compound that affects insect chordotonal organs, was also detected in samples only from hives kept in the Imperial Valley and at concentrations ranging from “trace” (<7 ppb) to 20 ppb. The following year only honey samples from the August sampling date were analyzed (Supplementary Table S10). DMPF was again found in all samples. Flonicamid was found in hives from citrus-avocado orchards in near Valley Center, CA, and one sample from the higher altitude hives but not in the AG hives. Methoxyfenozide, an insect growth regulator, was also detected in one of the CA samples.

Wax samples in August also had a high compound diversity (Table 5) as well as very high concentrations of certain compounds, particularly, but not exclusively, miticides. One wax sample from August 2016 had a DMPF concentration of 39,900 ppb, which was the highest concentration of any compound in any matrix in this study (Supplementary Table S11). In those same samples, fluvalinate ranged from 450 to 3720 ppb and coumaphos from 69–370 ppb. Two insecticides, fenpyroximate (up to 98 ppb) and phenothrin (up to 1900 ppb), were both found in all wax samples from that date. Permethrin was found in several samples across all original landscapes at up to 2430 ppb. Trifluralin, an herbicide, was found in most samples at up to 189 ppb. In the August 2017 samples fenpyroximate (up to 98 ppb) and permethrin (up to 1650 ppb) were found in all samples (Supplementary Table S12). Analysis of agrochemical diversity showed that there was no interaction between year and original landscape (P = 0.096) and in the main effects model that original landscape was not significant (P = 0.096).

**Table 5:** Number of agricultural compounds by type reported from wax and beebread for 2 apiaries per original landscape across 2 years, and beebread hazard quotients.

Matrix	Date	Agrochemical	AG 1^st^	AG 2^nd^	CA 1^st^	CA 2^nd^	MT 1^st^	MT 2^nd^
Wax	Aug 2016	Fungicides	1	1	1	1	3	1
		Herbicides	1	1	1	1	0	2
		Insecticides	5	7	4	4	5	4
		Miticides	4	4	4	4	4	4
		Total	11	13	10	10	12	11
Wax	Aug 2017	Fungicides	3	4	4	5	4	3
		Herbicides	3	2	2	2	1	2
		Insecticides	7	11	8	13	6	5
		Miticides	4	4	4	4	4	3
		Total	17	21	18	24	15	13
Beebread	Aug 2016	Fungicides	0	0	2	3	1	1
		Herbicides	2	1	0	0	0	0
		Insecticides	10	8	2	2	2	2
		Miticides	3	3	4	3	3	3
		Total	15	12	8	8	6	6
		Hazard quotient	1902	1128	4	3	4	1
Beebread	Aug 2017	Fungicides	0	2	3	5	1	0
		Herbicides	3	3	3	2	2	1
		Insecticides	6	8	3	8	4	3
		Miticides	1	3	2	2	2	3
		Total	10	16	11	17	9	7
		Hazard quotient	16	361	4	2	1111	2
			**AG 1**^**st**^ **+ AG 2**^**nd**^		**CA 1**^st^** + CA 2**^nd^		**MT 1**^st^** + MT 2**^nd^	
Beebread	Oct 2016	Fungicides	1		1		0	
		Herbicides	1		0		0	
		Insecticides	8		2		2	
		Miticides	4		4		3	
		Total	14		7		5	
		Hazard quotient	37		4		3	
			**AG 1**^st^	**AG 2**^nd^	**CA 1**^st^	**CA 2**^nd^	**MT 1**^st^	**MT 2**^nd^
Beebread	Oct 2017	Fungicides	3	6	0	0	2	4
		Herbicides	2	0	2	1	1	5
		Insecticides	3	3	3	2	3	3
		Miticides	0	2	0	1	2	2
		Total	8	11	5	4	8	14
		Hazard quotient	9	9	2	2	8	212

In October 2016 samples were pooled between apiaries within original landscape so results for just that level is reported. “1^st^” and “2^nd^” refer to the two apiaries within a given landscape for a given year. The synergists piperonyl butoxide and MGK264 were included as “insecticides” for counting purposes.

August, October and March beebread samples in both years were analyzed. The number of compounds detected in beebread prior to almond pollination was high compared to honey (Table 4). More insecticides were detected in the AG hives than in samples from any other sites, with the exception of one sample from CA in 2017–18, and more fungicides and herbicides were detected in CA and MT hives than those from the AG hives. Methoxyfenozide was detected in the most samples and at the highest concentration (350 ppb) in 2016–17 (Supplementary Table S13). The August 2017–18 sample from the MT 4 site had very high levels (2220 ppb) of cyphenothrin, a pyrethroid insecticide (Supplementary Table S14), and samples from the AG hives on that date had high methoxyfenozide concentrations, up to 1520 ppb, as that original landscape did the previous year. Also in 2017–18 both MT and AG samples had low levels (4–7 ppb) of diethyltoluamide (DEET, an insect repellant). Samples from October 2017 showed high levels of the fungicide chlorothalonil at 398–908 ppb among the AG3, AG4, MT3 and MT4 sites, with none reported from CA3 or CA4 (Supplementary Table S15). The MT3 sample also had 536 ppb of prodiamine, an herbicide not found in any other samples. An ANCOVA analysis of agrochemical diversity for the August samples, with “year” as the covariate, showed that original landscape was significant (P = 0.039). Post hoc contrasts indicated that the AG sites had significantly more kinds of compounds (13.3 ± 1.4) than the MT sites (7 ± 0.7) (no other contrasts were significant).

Samples from colonies sent to almonds had both a high diversity of compounds as well as high concentrations of certain compounds (Supplementary Table S16). Samples from March 2017 had pyriproxyfen (an insect growth regulator) at 480 and 870 ppb, as well as chlorpyrifos at 31–83 ppb and the fungicide cyprodinil at up to 48 ppb. Samples from almonds in March 2018 had the fungicide chlorothalonil at 250 to 6860 ppb and the herbicide pendimethalin at 110 to 132 ppb, as well as chlopyrifos in one sample at 287 ppb (Supplementary Table S17). With respect to agrochemical diversity for the March samples, there was a significant interaction between year and original landscape (P < 0.001); main effects of year and original landscape were also significant (P < 0.001 for both). A higher diversity of agrochemicals was observed in 2018 compared to 2017, and in almonds compared to blueberries (Table 6).

**Table 6:** Number of agricultural compounds by type reported from beebread collected in March, after almond pollination was completed, for each year of the study, and associated hazard quotients.

Sample date	Agrochemical	Almond			Blueberry		
		AG	CA	MT	AG	CA	MT
March 2016	Fungicides	4	4	6	1	0	1
	Herbicides	1	1	1	1	1	1
	Insecticides	6	4	3	2	0	1
	Miticides	3	2	2	2	4	3
	Total	14	11	12	6	5	6
	Hazard quotient	1462	575	1271	500	1	502
March 2017	Fungicides	11	11	12	0	1	2
	Herbicides	4	4	3	2	1	2
	Insecticides	4	4	5	0	1	0
	Miticides	1	1	1	2	2	2
	Total	20	20	21	4	5	6
	Hazard quotient	17	18	4932	5	5	5

Column headings refer to original landscapes.

## Discussion

In this study commercial honey bee colonies spent several months in apiaries in an “original landscape”, after which they were moved to a common site. If the original landscape had a short, limited impact on colony growth and behavior, then colonies would be expected to exhibit roughly the same behaviors among themselves after having been moved to the common landscape, or shortly thereafter, irrespective of the original landscape. After several months in the common site, half the colonies were moved to almond pollination and half remained at the common blueberry pollination site. Our goal was to monitor colonies during a typical commercial migration among sites and observe how colonies reacted and adapted to these changes.

The FOB visual assessment detected landscape-level differences among colonies only for the first sampling occasion, in August, across the two years of the study. Colonies from MT sites were, on average, about 10% larger than those from the AG group based on the FOB data. Brood levels were significantly higher among colonies from MT sites than those from the AG sites in August but the opposite was true by October. Average within-hive temperatures were slightly but consistently higher, and temperature amplitudes lower, in the AG colonies than either the CA or the MT colonies until mid-January. However, significant landscape effects on temperature amplitude were only detected starting mid-September (and continuing, with a few exceptions, for each time point until January) and temperature averages starting the end of October until January.

By mid-March no differences were observed between colonies in blueberries and those in almonds pollination with respect to either FOB or brood levels. Average hive temperatures were significantly different between the two pollination sites, but the direction of the difference changed between the two years, suggesting that it may have been due to weather or other environmental factors. The list of potential factors that might give rise to observed differences among the original landscape groups was large and investigative resources limited, so the study focused on agrochemical residues in honey, beebread and wax, and Varroa mite density.

Hive weight data were consistent between years, with hives in almonds gaining weight both years and those in blueberries losing weight in both years. Both the start and end of daily activity were significantly earlier for colonies in almonds compared to those in blueberries, but that was likely related to physical characteristics of the apiaries and how the hives were placed. The direction a hive is facing can affect the timing of the daily activity period^25^. Much of the hive weight gain during these periods was probably due to nectar and pollen collection. Hive weight loss at night during a nectar and pollen flow has been attributed to moisture loss of the drying nectar and pollen^22^. Hives in almonds lost on average more than 12 g per h at night while hives in blueberries lost on average less than 7 g per h, which suggests that, assuming the water content of the collected nectar was similar between sites, there was more material to dry in the hives in almonds.

Initial foraging populations were estimated by subtracting the estimated weight loss due to drying from the total hive weight change during the period of initial forager departure in the morning. These forager populations were significantly smaller for colonies placed in almonds compared to those in the blueberry environment, in spite of the higher weight gain for those colonies, which may have been due to a number of factors. One factor is the high density of forage in the almond environment – the colonies in almonds may have been able to maintain a higher foraging success with a smaller forager population compared to colonies in blueberries because of the high density of forage in almonds and/or because almond pollen is highly preferred compared to pollen in the blueberry site. Also, these estimates were made from changes in hive mass over time. It is also possible that forage was much more proximal in almonds, and surfeited foragers started returning as other foragers were still making their initial departure, confounding departures with returns and resulting in an underestimate of the forager population.

Mite levels were low overall and not significantly different among original landscapes, indicating that differences in mite densities were unlikely to explain differences among treatment groups. Varroa mite infestation is strongly correlated with some important viral diseases^26^ – we assumed a high incidence of at least Varroa-transmitted viral diseases was less likely when mite levels are as low as they were observed here. However, other diseases could also have been differentially distributed.

Pesticide residues were measured as composite samples on the level of the apiary or landscape. Honey samples were found to have few compounds – mainly DMPF, a by-product of the miticide amitraz, and flonicamid, an insecticide. Comb wax, which was only analyzed for the August samples in both years, typically had several compounds at comparatively high levels (>500 ppb). However, the relationship of residue concentrations in wax and bee health has not been firmly established, at least for many of the compounds detected. Wax, being lipophilic, can trap and store compounds, which may remain for years with little exposure to light, moisture or microbial activity. Because the compounds may be embedded in the wax matrix, the exposure of bees to those compounds may also be limited. Averaging across original landscapes and years, colonies kept in the AG and CA sites had on average 15.5 compounds per wax sample, while those in MT sites had on average 12.8 compounds, which is consistent with land use patterns.

Beebread may be the most revealing matrix for estimating the concentrations and diversity of agrochemicals bees are exposed to at a given point in time^6^. A recent study found that among 6 geographically disparate apiaries in the US, 79 different pesticides and metabolites were observed, with up to 10 distinct modes of action^27^. While wax and honey can last for many months or even years, bees prefer pollen <72 h old^28^, so it would more likely reflect the hive’s recent environment than wax or honey. In both August and October, beebread samples from the AG sites had more compounds (13.3 and 11.8, respectively) than either those in CA (11 and 5.8, respectively) or MT landscapes (7 and 8, respectively). These compounds included neonicotinoids, such as imidacloprid, and insect growth regulators (IGRs), such as methoxyfenozide, both of which have been shown to affect thermoregulation in bee colonies^8,25^. Some compounds, such as methoxyfenozide and flupyradifurone, were at high, albeit sublethal levels^29^. The IGRs methoxyfenozide and pyriproxyfen have been shown to reduce forager survival^4^. By October fewer pesticides were being detected overall, but the hives in MT landscapes were clearly being exposed to high levels of fungicides (chlorothalonil), herbicides (chlorthal-dimethyl and prodiamine) and IGRs (methoxyfenozide). Bee colonies were exposed to considerably more different compounds in almond pollination (16.3, averaging across all landscapes and across both years) than in blueberry pollination (5.3) at that time of year.

Agrochemical diversity is an important aspect of the in-hive pesticide exposome – greater compound diversity increases the possibility of interactions among compounds, which may be significant^6,30,31^. However, compound diversity alone is not likely to be a good measure of risk to bees. The pesticide hazard quotient was developed to take into account compound toxicity and residue concentration^5,6^. Hazard quotients calculated here showed high values (values >1000 are considered “high”^6^) for colonies in agricultural areas in the first year, but lower quotients for the same locations in the second year. In October quotients were low overall. Two mechanisms for lower agrochemical concentrations in the composite samples are: (1) bee colonies had largely consumed the pollen collected in the original landscape, given their preference for freshly-collected pollen, and replaced it with pollen from the new site; and (2) agrochemicals had decomposed perhaps through microbial activity. The second mechanism is unlikely, as microbial cell densities in bee bread are generally very low and static, even decreasing over time^32^. Some samples from the colonies kept in non-agricultural areas (MT) showed very high quotients due to high levels of particular compounds, suggesting the bees in at least some colonies may be been subjected to lethal levels. For samples collected in March, the composite bee bread sample from colonies used for almond pollination had high levels the first year, particularly compared to samples from colonies that remained in blueberries, but much less so the second year. No unusual bee mortality, such as masses of dead adult bees, was noted in any yards at any time.

Another approach would be to consider where and when compounds of known toxicity to honey bees were detected (Table 7). Emamectin benzoate and pyridaben are both comparatively toxic to honey bees but both those compounds were only detected in samples collected in March 2017 (Emamectin benzoate from colonies in blueberries and pyridaben from colonies in almonds) and thus their distribution has little explanatory value. Permethrin, found only in wax samples, is also somewhat toxic to bees^29,33^ but it was reported from at least one sample from all original landscapes in both years, so its occurrence would not explain landscape differences. A fourth example, chlorpyrifos, is comparatively toxic to bees^29,33^ and has been found to affect learning behavior at sublethal concentrations^34^. Chlorpyrifos was detected in beebread in August (both years) only from colonies in the AG group, and again in samples taken in March only from colonies used for almond pollination. Colonies from the AG sites had the highest average brood temperatures and lowest temperature variability, and colonies from almond pollination had the highest daily weight gain, both of which are generally considered positively correlated with colony health.

**Table 7:** Insecticides detected in hive matrices in order of acute contact LD_50_ obtained from the Pesticide Properties Database (https://sitem.herts.ac.uk/aeru/ppdb/en/atoz.htm) unless otherwise noted.

Compound	Type	Contact LD_50_ ug/bee	2016–17			2017–18			Total
			Honey (18)	Wax (6)	Bee bread (15)	Honey (6)	Wax (6)	Bee bread (18)	
Emamectin benzoate	Bacterial toxin	^b^0.002			2				**2**
Permethrin	Pyrethroid	0.024		3			6		**9**
Pyridaben	Pyridazinone	0.024			2				**2**
Chlorpyrifos	OP	0.059			5		5	2	**12**
Indoxacarb	Oxadiazine	0.08			2		2		**4**
Imidacloprid	Neonicotinoid	0.081			3				**3**
Carbaryl	Carbamate	0.14					1		**1**
Phenothrin	Pyrethroid	^a^>0.16		6					**6**
Tetramethrin	Pyrethroid	^a^>0.16					1		**1**
Malathion	OP	0.16		5			6		**11**
Spinosad	Bacterial toxin	^b^0.168			2			1	**3**
Cyhalothrin	Pyrethroid	^c^0.183			2				**2**
Cyphenothrin	Pyrethroid	^a^2.0						3	**3**
(V) Fluvalinate	Pyrethroid	4.32		6	14		5	3	**28**
(V) Coumaphos	OP	5.93		6	6		6		**18**
DDE p,p’	OC	6.4			5		1	1	**7**
Endosulfan II	OC	^c^7.05			1				**1**
Acetamiprid	Neonicotinoid	8.09			2			1	**3**
Bifenazate	Hydrazine carboxylate	8.5						1	**1**
Fenpyroximate	Pyrazolium	15.8		6			6	3	**15**
Propargite	Sulphite ester	47.9		4	1		5		**10**
Flonicamid	Pyridine	^c^71.2	6	2	3	3	2	3	**19**
Pyriproxyfen	IGR	74			2			3	**5**
Diflubenzuron	IGR	>74.2			3		4	2	**9**
(V) DMPF	Amitraz	75	18	6	15	6	6	18	**69**
Chlorantraniliprole	Anthranilic diamide	>100						10	**10**
Methoxfenozide	IGR	>100		1	11	1	3	12	**28**
Spirotetramat	Tetramic acid	>100						1	**1**
Hexythiazox	Carboxamide	^c^156						4	**4**
Buprofezin	IGR	>200						4	**4**
Etoxazole	Organofluorine	>200						2	**2**
Flupyradifurone	Butenolide	>200			1			5	**6**
Spirodiclofen	Tetronic acid	>200						1	**1**
Tebufenozide	IGR	>234			3		2	3	**8**
(V) Thymol	Plant phenol	NA		6	11		6	11	**34**
DEET	Repellant	NA					3	4	**7**

Honey, Wax and Beebread (total no. samples) show number of samples in which compound was detected. (V) indicates compound is used for Varroa mite control. OP = Organophosphate; OC = Organochlorine; IGR = Insect growth regulator.

^a^Unknown mode of exposure; ^b^modified from^33^; ^c^^36^; ^d^^6^; NA = not available.

The observed relationship between chlorpyrifos exposure and colony thermoregulation and weight gain suggests several non-exclusive possibilities, three of which are listed here. Some colony effects may be due to hormesis, defined as a change in the shape of the dose-response curve at low, sublethal concentrations of toxic compounds^35^. Compounds may cause negative effects at higher concentrations but not at low concentrations. Another possibility is that the LD_50_ may also be a poor measure of the impact of a compound on colony-level behavior. Several researchers have explored the use of hazard^36^ or risk quotients^37^ that take into account acute toxicity as well as time of exposure, insect life stage most at risk of exposure, application method and other factors (e.g., https://www.epa.gov/pesticide-science-and-assessing-pesticide-risks/models-pesticide-risk-assessment). Also, some kinds of exposure may affect honey bee behavior without affecting survivorship. Imidacloprid, which has a comparatively low LD_50_^25,33^, had measurable effects on colony behavior at concentrations that had no apparent effect on adult bee longevity^8^ raising the question of whether there are compounds with higher LD_50_ values but that also affect colony behavior. Incorporating colony-level behavior studies when evaluating novel pesticides may need to be considered.

A third possible explanation is that the observed distribution of vigorous colonies was not due to agrochemical exposure but rather to other factors such as ambient temperature or food quantity and/or quality. Colonies in the AG sites had greatest access to alfalfa fields; exposure to alfalfa has been associated with lower colony strength in the long term but positive colony growth in the short term^36^ and those colonies would also have had access to non-crop plants, particularly along irrigation canals and drainage ditches. Even together on the blueberry farm, colonies may still have had variable diets. Bee colonies in commercial blueberry fields in Canada have been found to have a low proportion of crop pollen compared to non-crop pollen and that beebread from those colonies has relatively low nutritional value compared to, for example, colonies placed in apple orchards^30^. If the bees did not prefer blueberry pollen they may have sought forage elsewhere. Finally, colonies in almond pollination gained weight while those in blueberry fields lost weight. Bee colonies in particular agricultural landscapes may thus thrive in spite of increased agrochemical exposure.

This work concerns a longitudinal study embedded in a commercial beekeeping operation, and was intended to monitor colony growth and activity in ways that would reveal differences among groups that strictly visual assessments may not detect. Such differences were observed and in the case of thermoregulation, those differences were largely consistent between the two years of the study. Colonies clearly adapt many aspects of their behavior and phenology to their current situation, but the effects of previous exposure to other environments can have be longer term and difficult to observe using strictly visual assessments. Owing to resource limitations (mainly sample processing costs), insufficient data was available to conclusively determine the cause of those differences among landscapes. Per hive analyses of agrochemicals and beebread nutritional quality, e.g., amino acid and essential fatty acid concentrations, and bee disease loads (especially for diseases not associated with Varroa) would have been very informative.

Which data in this study were the most valuable, and which data were the best value, taking into account the cost in terms of time and money resources? With respect to measures of colony health, estimating frames of bees was the least expensive, taking usually less than a minute and little cost other than transportation, but was also the least precise and revealed few differences among treatments. Any differences would probably have to be large before they could be detected. Measurement of brood surface area using frame photographs, taken during the visual assessment, had a comparatively low monetary cost (inexpensive cameras can suffice, and the image analysis software was free) and photographing frames can be fairly rapid, but analysis of the resulting photographs was time consuming. Visiting each of 60 hives once could generate 500–800 photographs (depending on the season) requiring on average a few minutes per photograph, and FOB and brood surface area data were collected with 2–4 months between data points. More frequent visits would increase transportation costs and frequency of colony disturbance. Continuous hive weight data showed significant treatment differences, in colony behavior, during a period when no other response variables did, and weight data is easy to collect without disturbing the colonies, easy to analyze and, for the most part, to interpret. However, the precision hive scales used here were expensive and cumbersome to install; less precise scales may be more convenient but may be less effective at detecting differences. Temperature data revealed many differences over a long period, the sensors are fairly inexpensive, and collection and analysis of the data are not time consuming. Further work is needed to improve understanding and exploitation of temperature data; changes in thermoregulation have been shown to be due to many factors. Clearly just one kind of data is unlikely to be sufficient in monitoring colony health.

## Materials and Methods

### Overall experimental design

In August of each of the two years of this study a total of 60 commercial hives were selected in two apiaries, each containing 60–80 hives, in each of three different landscapes: field crop and forage agriculture (AG) in the Imperial Valley (Holtville, CA), citrus and avocado groves (CA) in mid-altitude areas (Escondido and Valley Center, CA) and natural forage at higher elevations (MT) (near Santa Ysabel and Boulevard, CA) (Table 8). Apiary sites were assessed using the National Agricultural Statistical Service (NASS) Cropscape web site (https://nassgeodata.gmu.edu/ CropScape) to obtain acreage estimates for all land use categories within an approximately 1.7 km radius of the apiary (see Supplementary File). The hives, each of which had two “deep” Langstroth boxes (approximately 40.6 × 50.5 × 24.4 cm), had been either assembled the previous May and provided with a new queen (using open-mated queens from a commercial source) or kept as full colonies from the previous year. All colonies in both years of the study were in their respective sites by the end of May.

**Table 8:** Coordinates and names of apiaries.

Year	Location	Landscape	Group	Alt.	Coordinates	
2016–17	**Holtville**	**Agriculture**	**AG1**	**12**	**32 °53**′**9.46**″**N**	**115 °21**′**15.45**″**W**
	**Holtville**	**Agriculture**	**AG2**	**−1**	**32 °47**′**16.15**″**N**	**115 °20**′**59.72**″**W**
	**Escondido**	**Citrus/Avocado**	**CA1**	**182**	**33 °6**′**19.91**″**N**	**117 °1**′**19.49**″**W**
	**Escondido**	**Citrus/Avocado**	**CA2**	**245**	**33 °7**′**9.62**″**N**	**117 °1**′**30.66**″**W**
	**Santa Ysabel**	**Mountain**	**MT1**	**851**	**33 °4**′**5.56**″**N**	**116 °43**′**23.44**″**W**
	**Santa Ysabel**	**Mountain**	**MT2**	**966**	**33 °5**′**14.21**″**N**	**116 °42**′**11.56**″**W**
	Valley Center	Blueberry	BLU	284	33 °18′21.71″N	117 °2′1.36″W
	Bakersfield	Almonds	ALM	113	35 °26′3.67″N	119 °10′24.61″W
	Escondido	Citrus	—	133	33° 5′41.91″N	116 °56′43.98″W
2017–18	**Holtville**	**Agriculture**	**AG3**	**−5**	**32 °53**′**4.15**″**N**	**115 °19**′**41.23**″**W**
	**Holtville**	**Agriculture**	**AG4**	**7**	**32 °50**′**24.48**″**N**	**115 °17**′**27.71**″**W**
	**Valley Center**	**Citrus/Avocado**	**CA3**	**543**	**33 °19**′**36.14**″**N**	**116 °57**′**55.76**″**W**
	**Valley Center**	**Citrus/Avocado**	**CA4**	**259**	**33 °19**′**30.02**″**N**	**116 °59**′**44.17**″**W**
	**Boulevard**	**Mountain**	**MT3**	**1046**	**32 °39**′**31.85**″**N**	**116 °17**′**0.66**″**W**
	**Boulevard**	**Mountain**	**MT4**	**1143**	**32 °41**′**42.89**″**N**	**116 °16**′**56.45**″**W**
	Valley Center	Blueberry	BLU	284	33 °18′21.71″N	117 °2′1.36″W
	Bakersfield	Almonds	ALM	113	35 °26′3.67″N	119 °10′24.61″W

“Alt.” = altitude in meters. Landscapes in bold show the “original landscape” locations.

Ten hives were selected in each of two apiaries at least 2 km apart within each landscape. Hives were at least 3 m apart within the original apiary and did not neighbor any other selected hives. Frames of bees (FOB) were visually estimated in both top and bottom boxes by the number of between-frame spaces and half spaces covered by bees when observed from above. The same member of the team (EB) estimated all FOB values. The same worker estimated FOB levels in another study in which adult bee masses were measured independently using another method (see^25^), and the linear regression between that worker’s FOB estimates and adult bee masses are provided (see Supplementary File). After visual estimation, both sides of all frames with brood were photographed using a 16.3 megapixel digital camera (Canon Rebel SL1, Canon USA, Inc., Melville, NY). The area of sealed brood per frame was estimated later from the frame photographs using either ImageJ software (version 1.47. W. Rasband, National Institutes of Health, USA), or CombCount;^38^ this method has been described in other publications (see^8,13,16,25^). Any colony that had capped brood was included in the study.

Samples of honey (approx. 2 ml per hive), beebread (from approx. 15 cells per hive) and comb wax (approx. 1 g) were collected. Adult bees (50–100) were collected from the outer brood nest (usually the first frame encountered with brood when working from one side of the box to the other) and stored on ice. Based on where the sample was collected, it was assumed to contain a high proportion of nurse bees with some foragers. A temperature sensor (iButton Thermochron, precision ±0.06 °C) enclosed in plastic tissue embedding cassettes (Thermo Fisher Scientific, Waltham, MA) was stapled to the center of the top bar on the 5th frame in the bottom box of each hive and set to record every 30 min. In October all hives were moved to a blueberry farm (Valley Center, CA) for commercial pollination. During September and early October all hives were given one or more supplemental soybean flour-based protein patties and a 50 g patty consisting of palm oil and 0.09% (w/w) amitraz to control Varroa mites.

In October all hives were assessed again in the same manner, and the temperature sensor was removed, replaced with a fresh sensor, and then downloaded. In December and January 2017 all hives were given protein patty and another patty containing amitraz. In January 2017 all hives were visited to 1) estimate the frames of bees; and 2) change the temperature sensor. Because photographing brood frames is an invasive procedure that requires removing the frames and shaking them free of bees, it was not conducted in January. Hives were randomly assigned to either remain in the blueberry farm or be sent to an almond orchard for commercial pollination. Because commercial pollination contracts stipulate the required size of the bee colonies (frames of bees as estimated by either the commercial beekeeper or another party), not all hive designated for almond pollination were sent to almond orchards. However, all hives designated to remain in the blueberry farm remained there through March. The apiary in the almond orchard contained only hives used in this study (about 30 hives).

During the almond pollination period, ten hives were selected from the group of hives sent to the almond orchard and ten selected from the group of hives that remained in the blueberries. Hives were placed on stainless steel electronic scales (Tekfa model B-2418 and Avery Weigh-Tronix model BSAO1824–200) (max. capacity: 100 kg, precision: ±20 g; operating temperature: −30 °C to 70 °C) and linked to 12-bit dataloggers (Hobo U-12 External Channel datalogger, Onset Computer Corporation, Bourne, MA, USA) with weight recorded every 15 minutes. The scales were powered by deep-cycle batteries connected to solar panels. All surviving hives in both groups were evaluated for a final time after the end of the almond pollination period.

Wax, beebread and honey samples from the August evaluation were pooled by apiary (i.e. 6 groups per year) and submitted to the Laboratory Approval and Testing Division, Agricultural Marketing Service, USDA (LATD), Gastonia, NC, for full panel testing (the number of compounds detected during the full panel testing varied from 200–214 compounds, including insecticides, fungicides, miticides, herbicides, synergists and breakdown products; please see Supplementary Tables for a complete list). Adult bee samples were stored at −20 °C and subjected to an alcohol wash to remove mites. Mites and bees were then counted to estimate the number of mites per 100 bees for each sampling date.

### Data analysis

Brood surface area and FOB were considered with respect to three sampling occasions: August, October and March. Data were analyzed using repeated measures MANOVA (Proc Glimmix, version 9.4, SAS Inc. 2002), with the original landscape factor and year as fixed factors and the August values of the response variables as covariates for the October and March analyses. Varroa density per 100 bees were analyzed with respect to original landscape, year and time of year as fixed factors. Varroa mite data were log transformed and subjected to a repeated-measures ANOVA with landscape, year and sampling occasion and interactions as fixed factors.

Internal hive temperature data were divided into daily average and within-day detrended data. Detrended data were calculated as the difference between the 25 hour running average and the raw data. Sine curves were fit, via least sums of squares (see^16^), to 3-day subsamples of detrended data taken sequentially by day, and curve amplitudes, representing estimates of daily hive temperature variability, were used as response variables. Repeated measures MANOVA was used to evaluate the effects of treatment, day, and their interaction, with the sealed brood surface area at the start of the experiment in August (both years) as a covariate on both the average daily hive temperature and the amplitudes of the fit sine curves. Thermoregulation is closely linked to brood production^16,17^ so it was used as a covariate to control for pre-existing differences among colonies. Temperature amplitude datasets were reduced to one value every 3 d for repeated measures analysis to ensure no overlap between subsamples. Temperature data were evaluated with respect to three time periods: mid-August to October, November to mid-January, and mid-January to beginning of March. Colonies were removed from the study once their death had been confirmed by visual assessment.

Continuous hive weight data were likewise considered with respect to daily weight change and within-day changes. Because of the low number of hives on scales per pollination site per year (10), original landscape groups were no longer considered. Daily weight change was calculated by averaging, for each 24 hour period, all the weight data for a given hive and subtracting that value from the value for the following day. These between-day weight changes were analyzed using repeated-measures MANOVA with pollination sites (almond or blueberry; hereafter “pollination”), year, and 2-way interactions as fixed effects and pre-pollination weight as a covariate.

Weight data were detrended for each day by subtracting the hive weight estimate at midnight (or closest time thereafter) from each subsequent weight value over the next 24 h (see^22^). The resulting within-day weight datasets were modeled using the “segmented” function in R (version 3.6.1. The R Foundation for Statistical Computing) which fits a segmented line derived from a linear or generalized linear model to a dependent variable using a bootstrapping procedure^39^. Piecewise regressions with 4 breakpoints were fit to the data, which yielded estimates for 10 parameters: 4 break point values, 5 slope values and the adjusted r^2^. Because the data were detrended by subtracting the raw data value at midnight, daily datasets were mathematically independent from each other. The following values were obtained:

1. Dawn break point (start of daily hive activity, usually the 1st break point);
2. Dusk break point (end of daily hive activity, usually the 4^th^ break point)
3. Slope of the first segment after dawn, usually the 2nd segment;
4. Night slope: the slope of the 1st segment, representing hive weight change during the night.

While daily patterns in hive weight change are generally consistent, they can also be variable. To reduce what were considered known errors, if the 1st break point occurred before 4 AM, it was assumed to be an error, the 2nd break point was used instead as the dawn break point (no restrictions were placed on that new estimate) and the 3rd, rather than 2nd, segment was assumed to be the first segment after dawn (value 3 in the list above). Likewise, if the 4th break point occurred after 8 PM it was assumed to be an error and the 3rd break point was taken as the dusk break point (again with no restrictions placed on that new estimate). The slope of the first segment after dawn was multiplied by the length of time between the break points on either end to estimate hive weight loss during that period. This hive weight loss was attributed to two factors: (1) moisture loss (nectar drying, respiration, etc.); and (2) forager departure. Weight loss due to forager departure was then calculated by (1) multiplying the slope of the first segment after dawn by the length of the projection of the segment on the X axis (i.e. time) to generate an estimate of total hive weight loss during that period, and then subtracting from that value the estimated weight loss due to drying:

1 $$\Delta {\rm{F}}={{\rm{S}}}_{{\rm{F}}}{({\rm{t}}}_{{\rm{D}}+1}-{{\rm{t}}}_{{\rm{D}}})-{{\rm{S}}}_{{\rm{N}}}{({\rm{t}}}_{{\rm{D}}+1}-{{\rm{t}}}_{{\rm{D}}})$$

where ΔF is the hive weight change due to forager departure, S_F_ is the slope of the piecewise regression segment associated with forager departure, t_D_ is the time of the dawn break point, t_D+1_ is the time of the following break point, and S_N_ is the slope of the first segment of the regression, representing hive moisture loss due to drying at night (this was used as a proxy to estimate the drying rate during forager departure). Dawn and dusk break points, S_N_ and ΔF were used as response variables in repeated-measures ANOVAs with treatment and year as fixed factors, hive as a random factor and January FOB as a covariate to control for colony size. Days with rainfall >3 mm were removed from the analysis because of the effect of rainfall on the shape of within-day hive weight changes.

Pesticide diversity data (i.e. the total number of compounds detected) in wax and beebread samples were analyzed within sampling occasion using ANCOVA, with year as the covariate and landscape as the main factor. First an interaction model was tested; if the interaction was not significant, slopes were assumed equal and a main effects model was tested. Because beebread samples in October 2016 were pooled across landscape, only August and March beebread data were statistically analyzed. Pesticide hazard quotients were calculated using residue data and information on honey bee LD_50_ levels obtained from either the University of Hertfordshire Pesticide Properties Database (https://sitem.herts.ac.uk/aeru/ppdb/en/atoz.htm), or from recent publications, i.e.^6,33,36^. Because “contact” LD_50_ data were more readily available than “oral” LD_50_, those data were used throughout. Hazard quotients were calculated by dividing the residue concentration in ppb by the LD_50_ value in µg per bee, and then summing the values for all detected compounds for a given sample^5,6^. In cases where the LD_50_ was presented as the lower end of a range of values, such as “>100 µg per bee” the inequality sign was simply removed, so the values shown here should be considered lower estimates. Where “trace” amounts of an agrochemical was reported, a token value of 1 ppb was used.

## Supplementary information

Supplementary information.

Supplementary information S2.

## Data Availability

All raw data on hive assessment, temperature and weight are available in the Supplementary File.
