# Supplementary material for: Landscape factors influencing honey bee colony behavior in Southern California commercial apiaries
Source: Sci Rep. 2020 Mar 19;10:5013. doi: 10.1038/s41598-020-61716-6 (PMC7081305; doi:10.1038/s41598-020-61716-6)
Supplement: Supplementary file 1 — Supplementary information. [file 41598_2020_61716_MOESM1_ESM.docx]

**Landscape factors influencing honey bee colony behavior in Southern California commercial apiaries**

**William G. Meikle*, Milagra Weiss, Eli Beren**

**S1 Table**. MANOVA results for brood surface area from August until mid March. Landscape (Agriculture, Citrus-avocado or Mountain) and Year (2016-17 and 2017-18) and Pollination site (almonds or blueberries) were fixed effects, hive number was a random factor and August brood levels were used as a covariate. AR(1) covariance model was used

| Date | Factor | Num DF | Den DF | F Value | Pr > F |
| --- | --- | --- | --- | --- | --- |
| Aug. | Original landscape | 2 | 113 | 12.77 | <0.0001 |
|  | Year | 1 | 113 | 10.89 | 0.0013 |
|  | Orig. landsc.*Year | 2 | 113 | 8.78 | 0.0003 |
| Oct. | Original landscape | 2 | 108 | 8.55 | 0.0004 |
|  | Year | 1 | 108 | 5.98 | 0.0161 |
|  | Orig. landsc.*Year | 2 | 108 | 2.42 | 0.0936 |
|  | August_brood | 1 | 108 | 1.39 | 0.2411 |
| March | Pollination | 1 | 92 | 1.18 | 0.2799 |
|  | Original landscape | 2 | 92 | 1.33 | 0.2698 |
|  | Year | 1 | 92 | 0.02 | 0.8876 |
|  | Pollination*Year | 1 | 92 | 0.65 | 0.4219 |
|  | Orig. landsc.*Year | 2 | 92 | 1.39 | 0.2551 |
|  | Poll.* Orig. landsc. | 2 | 92 | 2.52 | 0.0864 |
|  | August_brood | 1 | 92 | 1.77 | 0.1871 |

**S2 Table**. MANOVA results for frames of bees (FOB) from August until mid March. Landscape (Agriculture, Citrus-avocado or Mountain), Year (2016-17 and 2017-18), Sample date and Pollination site (almonds or blueberries) were fixed effects, hive number was a random factor and August FOB levels were used as a covariate. AR(1) covariance model was used

| Date | Factor | Num DF | Den DF | F Value | Pr > F |
| --- | --- | --- | --- | --- | --- |
| Aug. | Original landscape | 2 | 114 | 3.67 | 0.0284 |
|  | Year | 1 | 114 | 2.27 | 0.1346 |
|  | Orig. landsc.*Year | 2 | 114 | 5.41 | 0.0057 |
| Oct.-Jan. | Original landscape | 2 | 98.14 | 3.02 | 0.0533 |
|  | Year | 1 | 98.11 | 2.35 | 0.1287 |
|  | Sample date | 1 | 97.05 | 28.27 | <0.0001 |
|  | Orig. landsc.*Year | 2 | 97.71 | 6.73 | 0.0018 |
|  | Orig. landsc.*Sample date | 2 | 97.01 | 5.59 | 0.0050 |
|  | August_FOB | 1 | 108.4 | 1.39 | 0.2412 |
| March | Pollination | 1 | 92 | 1.72 | 0.1927 |
|  | Original landscape | 2 | 92 | 0.92 | 0.4004 |
|  | Year | 1 | 92 | 2.40 | 0.1244 |
|  | Pollination*Year | 1 | 92 | 0.41 | 0.5237 |
|  | Orig. landsc.*Year | 2 | 92 | 3.65 | 0.0299 |
|  | Poll.* Orig. landsc. | 2 | 92 | 0.13 | 0.8756 |
|  | August_FOB | 1 | 92 | 1.60 | 0.2085 |

**S3 Table**. MANOVA results for hive temperature data for two time periods: mid August to end of October, and beginning of November to mid January. Landscape (Agriculture, Citrus-almond or Mountain) and Day were fixed effects, hive number was a random factor and August brood level was a covariate.

| Response variable | Date | Factor | Num DF | Den DF | F | Pr > F |
| --- | --- | --- | --- | --- | --- | --- |
| Average temperature | Aug.-Oct. | Original landscape | 2 | 70.08 | 1.72 | 0.1857 |
| (log transformed) |  | Day | 86 | 8729 | 3.41 | <0.0001 |
|  |  | Year | 1 | 73.43 | 1.56 | 0.2159 |
|  |  | Orig. landsc.*Day | 172 | 9049 | 1.52 | <0.0001 |
|  |  | Orig. landsc.*Year | 2 | 73.45 | 0.27 | 0.7644 |
|  |  | August_brood | 1 | 72.53 | 0.26 | 0.6110 |
|  | Nov.-Jan. | Original landscape | 2 | 168.7 | 10.97 | <0.0001 |
|  |  | Day | 75 | 7666 | 20.28 | <0.0001 |
|  |  | Year | 1 | 175 | 0.27 | 0.6037 |
|  |  | Orig. landsc.*Day | 150 | 7756 | 4.52 | <.0001 |
|  |  | Orig. landsc.*Year | 2 | 175 | 1.06 | 0.3471 |
|  |  | August_brood | 1 | 173.3 | 1.57 | 0.2122 |
| Temperature amplitude | Aug.-Oct. | Original landscape | 2 | 141.3 | 4.95 | 0.0084 |
| (log transformed) |  | Day | 28 | 2901 | 16.24 | <0.0001 |
|  |  | Year | 1 | 142.9 | 0.01 | 0.9166 |
|  |  | Orig. landsc.*Day | 56 | 3020 | 6.53 | <0.0001 |
|  |  | Orig. landsc.*Year | 2 | 143 | 0.31 | 0.7352 |
|  |  | August_brood | 1 | 141.7 | 6.61 | 0.0112 |
|  | Nov.-Jan. | Original landscape | 2 | 115.4 | 8.82 | 0.0003 |
|  |  | Day | 22 | 2199 | 26.66 | <0.0001 |
|  |  | Year | 1 | 108.5 | 3.49 | 0.0644 |
|  |  | Orig. landsc.*Day | 44 | 2281 | 2.50 | <0.0001 |
|  |  | Orig. landsc.*Year | 2 | 108.5 | 0.42 | 0.6554 |
|  |  | August_brood | 1 | 108.5 | 1.16 | 0.2830 |

**S4 Table**. MANOVA results for hive temperature data during spring pollination. Pollination environment (almonds or blueberries) and Day were fixed effects, hive number was a random factor and August brood levels were used as a covariate.

| Response variable | Factor | Num DF | Den DF | F Value | Pr > F |
| --- | --- | --- | --- | --- | --- |
| Average daily temperature | Pollination | 1 | 136.5 | 4.18 | 0.0429 |
| (log transformed) | Original landscape | 2 | 136.2 | 1.56 | 0.2138 |
|  | Day | 55 | 4925 | 21.39 | <0.0001 |
|  | Year | 1 | 138.3 | 25.30 | <0.0001 |
|  | Poll.* Orig. landsc. | 2 | 135.4 | 0.74 | 0.4771 |
|  | Poll.*Day | 55 | 4925 | 4.25 | <0.0001 |
|  | Poll.*Year | 1 | 138.4 | 1.55 | 0.2158 |
|  | Orig. landsc.*Day | 110 | 5012 | 1.09 | 0.2396 |
|  | Orig. landsc.*Year | 2 | 138.3 | 0.83 | 0.4377 |
|  | August_brood | 1 | 136.1 | 0.62 | 0.4321 |
| Temperature amplitude | Pollination | 1 | 125.5 | 1.21 | 0.2742 |
| (log transformed) | Original landscape | 2 | 124.9 | 0.88 | 0.4186 |
|  | Day | 19 | 1680 | 30.54 | <0.0001 |
|  | Year | 1 | 130.3 | 64.89 | <0.0001 |
|  | Poll.* Orig. landsc.. | 2 | 124.3 | 1.36 | 0.2602 |
|  | Poll.*Day | 19 | 1679 | 3.13 | <0.0001 |
|  | Poll.*Year | 1 | 130.5 | 4.17 | 0.0432 |
|  | Orig. landsc.*Day | 38 | 1687 | 0.83 | 0.7567 |
|  | Orig. landsc.*Year | 2 | 130.2 | 0.75 | 0.4725 |
|  | August_brood | 1 | 125.7 | 0.71 | 0.3996 |

**S5 Table**. MANOVA results for daily hive weight change during almond and blueberry pollination. Hives in almond pollination were placed near Bakersfield, CA, while hives in blueberry pollination were placed near Escondido, CA.

| Response variable | Factor | Num DF | Den DF | F Value | Pr > F |
| --- | --- | --- | --- | --- | --- |
| Daily hive weight gain | Pollination | 1 | 249.3 | 172.29 | <0.0001 |
|  | Day | 31 | 721.2 | 18.07 | <0.0001 |
|  | Year | 1 | 241 | 11.39 | 0.0009 |
|  | Pollination*Day | 31 | 721.2 | 12.55 | <0.0001 |
|  | Pollination*Year | 1 | 242.2 | 0.09 | 0.7628 |
|  | Initial hive weight | 1 | 232.9 | 3.94 | 0.0483 |

**S6 Table**. MANOVA results for the effects of treatment on dawn and dusk break points, and forager population mass, for piecewise regressions fit to continuous weight data from the 2017 almond pollination. Analyses done with an AR(1) covariance matrix.

| Response variable | Factor | Num DF | Den DF | F Value | Pr > F |
| --- | --- | --- | --- | --- | --- |
| Dawn break point | Pollination | 1 | 301.3 | 25.78 | <0.0001 |
| t_D_ | Day | 34 | 880.3 | 6.88 | <0.0001 |
|  | Year | 1 | 290.1 | 3.23 | 0.0732 |
|  | Pollination*Day | 32 | 876.9 | 8.35 | <0.0001 |
|  | Pollination*Year | 1 | 292.4 | 0.04 | 0.8482 |
|  | FOB in January | 1 | 287 | 0.31 | 0.5774 |
| Dusk break point | Pollination | 1 | 300.4 | 13.97 | 0.0002 |
|  | Day | 34 | 879.7 | 8.42 | <0.0001 |
|  | Year | 1 | 289.1 | 9.08 | 0.0028 |
|  | Pollination*Day | 32 | 876.3 | 7.92 | <0.0001 |
|  | Pollination*Year | 1 | 291.4 | 4.11 | 0.0434 |
|  | FOB in January | 1 | 286 | 0.51 | 0.4740 |
| Night weight change rate | Pollination | 1 | 178.5 | 50.62 | <0.0001 |
| S_N_ | Day | 32 | 768.9 | 14.66 | <0.0001 |
|  | Year | 1 | 176.3 | 11.82 | 0.0007 |
|  | Pollination*Day | 27 | 762.7 | 12.74 | <0.0001 |
|  | Pollination*Year | 1 | 178.7 | 0.74 | 0.3893 |
|  | FOB in January | 1 | 169.6 | 7.67 | 0.0063 |
| Forager population mass | Pollination | 1 | 210.6 | 47.55 | <0.0001 |
| ΔF | Day | 32 | 768.1 | 5.97 | <0.0001 |
|  | Year | 1 | 204.8 | 0.03 | 0.8620 |
|  | Pollination*Day | 27 | 762.1 | 7.33 | <0.0001 |
|  | Pollination*Year | 1 | 207.6 | 1.24 | 0.2660 |
|  | FOB in January | 1 | 196.2 | 3.84 | 0.0516 |

**S7 Table**. MANOVA results for Varroa density (Varroa mites per 100 bees) from samples collected in August and October in 2016 and 2017. Landscape (Agriculture, Citrus-almond or Mountain), Year (2016 or 2017) and Sampling occasion (August or October) were fixed effects, and hive number was a random factor.

| Factor | Num DF | Den DF | F Value | Pr > F |
| --- | --- | --- | --- | --- |
| Original landscape | 2 | 111.3 | 0.57 | 0.5672 |
| Year | 1 | 111.3 | 53.65 | <0.0001 |
| Sampling occasion | 1 | 113.8 | 0.01 | 0.9286 |
| Orig. landsc.*Year | 2 | 111.3 | 1.48 | 0.2316 |
| Orig. landsc.*Sampling occasion | 2 | 113.8 | 5.03 | 0.0081 |

**S8** Table. Agrochemicals detected in all matrices (bee bread, wax, honey) over both years of the study.

| Agrochemical | Type |  | Agrochemical | Type |
| --- | --- | --- | --- | --- |
| Azoxystrobin | Fungicide |  | Acetamiprid (neonic) | Insecticide |
| Boscalid | Fungicide |  | Bifenazate | Insecticide |
| Carbendazim (MBC) | Fungicide |  | Buprofezin | Insecticide |
| Chlorothalonil | Fungicide |  | Carbaryl | Insecticide |
| Cyprodinil | Fungicide |  | Chlorantraniliprole | Insecticide |
| Difenoconazole | Fungicide |  | Chlorpyrifos | Insecticide |
| Fenamidone | Fungicide |  | Cyhalothrin total | Insecticide |
| Fludioxonil | Fungicide |  | Cyphenothrin | Insecticide |
| Fluopyram | Fungicide |  | DDE p,p’ (DDT product) | Insecticide |
| Fluxapyroxad* | Fungicide |  | Diflubenzuron (IGR) | Insecticide |
| Metalaxyl | Fungicide |  | Emamectin Benzoate | Insecticide |
| Penthiopyrad* | Fungicide |  | Endosulfan II | Insecticide |
| Propamocarb hydrochloride* | Fungicide |  | Etoxazole | Insecticide |
| Propiconazole | Fungicide |  | Fenpyroximate | Insecticide |
| Pyraclostrobin | Fungicide |  | Flonicamid | Insecticide |
| Pyrimethanil | Fungicide |  | Flupyradifurone | Insecticide |
| Tebuconazole | Fungicide |  | Hexythiazox | Insecticide |
| Trifloxystrobin | Fungicide |  | Imidacloprid (neonic) | Insecticide |
| Acetochlor | Herbicide |  | Indoxacarb | Insecticide |
| Atrazine | Herbicide |  | Malathion | Insecticide |
| Bensulide* | Herbicide |  | Methoxyfenozide (IGR) | Insecticide |
| Chlorthal-dimethyl (DCPA)* | Herbicide |  | Permethrin* | Insecticide |
| Diuron | Herbicide |  | Phenothrin | Insecticide |
| Fluometuron | Herbicide |  | Propargite | Insecticide |
| Metolachlor | Herbicide |  | Pyridaben | Insecticide |
| Pendimethalin | Herbicide |  | Pyriproxyfen (IGR) | Insecticide |
| Pronamide | Herbicide |  | Spinosad | Insecticide |
| Simazine | Herbicide |  | Spirodiclofen | Insecticide |
| Trifluralin | Herbicide |  | Spirotetramat | Insecticide |
| Coumaphos | Miticide |  | Tebufenozide (IGR) | Insecticide |
| DMPF | Miticide |  | Tetramethrin | Insecticide |
| Fluvalinate | Miticide |  | DEET* | Repellant |
| Thymol | Miticide |  | MGK-264 | Synergist |
|  |  |  | Piperonyl butoxide | Synergist |

**Honey samples**

S9 Table. Agrochemical residues in honey for 2016-17 samples. LOD means Limit of Detection. The lab where the samples were processed improved some of their methods so the LOD values for the 2017-18 samples were often lower than those for the 2016-17 samples. However, in a few cases the LOD values were revised upward. 2,4 Dimethylphenyl formamide (DMPF) is a breakdown product of amitraz. Flonicamid has been classified as a neonicotinoid pesticide.

| Sample date: | Apiary group (location) | 2,4 Dimethylphenyl formamide (DMPF); LOD: 5 | Flonicamid  LOD: 7 |
| --- | --- | --- | --- |
| Aug 2016 | AG1 | 16 | Trace |
|  | AG2 | 9 | 20 |
|  | CA1 | 59 | - |
|  | CA2 | 60 | - |
|  | MT1 | 28 | - |
|  | MT2 | 73 | - |
| Oct. 2016 | AG1 + AG2 (BLU) | 68 | Trace |
|  | CA1+ CA 2 (BLU) | 155 | - |
|  | MT1 + MT2 (BLU) | 81 | - |
| Feb. 2017 | AG1 + AG2 (BLU ) | 74 | Trace |
|  | CA1 + CA2 (BLU ) | 145 | - |
|  | MT1 + MT2 (BLU) | 110 | - |
| March 2017 | AG1 + AG2 (BLU) | 6 | Trace |
|  | AG1 + AG2 (ALM) | 80 | Trace |
|  | CA1 + CA2 (BLU) | 97 | - |
|  | CA1 + CA2 (ALM) | 121 | - |
|  | MT1 + MT2 (BLU 1) | 115 | - |
|  | MT1 + MT2 (ALM) | 35 | - |

**Honey samples cont’d**

S10 Table. Agrochemical residues in honey for August 2017 samples. Methoxyfenozide is an Insect Growth Regulator (IGR).

|  | DMPF | Flonicamid | Methoxyfenozide |
| --- | --- | --- | --- |
| Hive Location | LOD: 1.5 ppb | LOD: 7 ppb | LOD: 1 ppb |
| AG3 | 40 | - | - |
| AG4 | 13 | - | - |
| CA3 | 7 | Trace | - |
| CA4 | 32 | Trace | Trace |
| MT3 | 20 | - | - |
| MT4 | 18 | Trace | - |

**Wax samples**

S11 Table. Agrochemical residues in wax samples from August 2016. Note the high concentrations of varroacides such as DMPF, fluvalinate, thymol, and coumaphos. Some of this comb may have been old – some compounds are very stable in the dark conditions inside a hive. F = fungicide; H = herbicide; I = insecticide; V = varroacide

| Agrochemical | Type | LOD | AG1 | AG2 | CA1 | CA2 | MT1 | MT2 |
| --- | --- | --- | --- | --- | --- | --- | --- | --- |
| DMPF | V | 5 | 1360 | 700 | 5430 | 950 | 715 | 39900 |
| Acetochlor | H | 15 | - | - | - | - | - | 26 |
| Carbendazim (MBC) | F | 5 | Trace | Trace | Trace | Trace | Trace | Trace |
| Chlorothalonil | F | 100 | - | - | - | - | Trace | - |
| Coumaphos | V | 3 | 71 | 245 | 69 | 150 | 105 | 370 |
| Coumaphos oxon | V | 2 | Trace | Trace | Trace | Trace | Trace | Trace |
| Fenpyroximate | I | 4 | 98 | 26 | 41 | Trace | 86 | 20 |
| Flonicamid | I | 15 | Trace | Trace | - | - | - | - |
| Fluvalinate | V | 5 | 3720 | 450 | 794 | 990 | 960 | 2410 |
| Malathion | I | 10 | 28 | 15 | 43 | 14 | - | 27 |
| Methoxyfenozide | I | 5 | - | Trace | - | - | - | - |
| Permethrin* | I | 25 | - | 2430 | - | 1400 | 963 | - |
| Phenothrin | I | 30 | 340 | 1380 | 1900 | 971 | 925 | 357 |
| Piperonyl butoxide** | I | 15 | - | - | Trace | - | Trace | - |
| Propargite | I | 15 | Trace | Trace | - | - | Trace | Trace |
| Propiconazole | F | 15 | - | - | - | - | 30 | - |
| Thymol | V | 50 | 264 | 102 | 173 | 491 | Trace | 176 |
| Trifluralin | H | 5 | 14 | 189 | 12 | 7 | - | 8 |

*Was not tested in the 2016-17 study

**Piperonyl butoxide, a synergist classified here as an insecticide

**Wax samples cont’d**

S12 Table. Agrochemical residues in wax samples from August 2017. F = fungicide; H = herbicide; I = insecticide; V = varroacide

|  |  |  | August 2017 | |  |  |  |  |  |  |
| --- | --- | --- | --- | --- | --- | --- | --- | --- | --- | --- |
| Agrochemical | Type | LOD | AG3 | AG4 |  | CA3 | CA4 |  | MT3 | MT4 |
| DMPF | V | 1.5 | 344 | 5310 |  | 890 | 2760 |  | 641 | 2830 |
| Atrazine | H | 4 | Trace | - |  | - | - |  | - | - |
| Carbaryl | I | 5 | - | - |  | - | Trace |  | - | - |
| Carbendazim | F | 2 | - | - |  | 3 | Trace |  | Trace | Trace |
| Chlorpyrifos | I | 5 | - | Trace |  | Trace | Trace |  | Trace | Trace |
| Chlorthal-dimethyl (DCPA) | H | 2 | Trace | Trace |  | Trace | Trace |  | Trace | Trace |
| Coumaphos | V | 4 | 96 | 84 |  | 112 | 173 |  | 86 | 95 |
| Coumaphos oxon | V | 0.5 | 8 | 7 |  | 4 | 8 |  | 5 | 2 |
| Cyprodinil | F | 2 | 7 | 98 |  | 3 | 12 |  | 3 | Trace |
| DDE p,p' | I | 2 | - | - |  | - | Trace |  | - | - |
| DEET* | I | 3 | - | 5 |  | 5 | - |  | 5 | - |
| Diflubenzuron | I | 2 | - | Trace |  | - | Trace |  | - | - |
| Fenamidone | F | 1 | - | Trace |  | - | - |  | - | - |
| Fenpyroximate | I | 3 | 8 | 11 |  | 17 | 98 |  | 67 | 67 |
| Flonicamid | I | 7 | - | 12 |  | - | Trace |  | - | - |
| Fluvalinate | V | 25 | 1160 | 503 |  | 1320 | 1790 |  | 1830 | - |
| Hexythiazox | I | 2 | Trace | 7 |  | Trace | Trace |  | - | - |
| Indoxacarb | I | 7 | Trace | Trace |  | - | - |  | - | - |
| Malathion | I | 25 | Trace | Trace |  | 94 | Trace |  | Trace | Trace |
| Methoxyfenozide | I | 1 | 14 | 17 |  | - | 9 |  | - | - |
| MGK-264** | I | 7 | - | - |  | - | 19 |  | - | - |
| Pendimethalin | H | 20 | Trace | Trace |  | Trace | Trace |  | - | Trace |
| Penthiopyrad* | F | 1 | Trace | Trace |  | 5 | Trace |  | 5 | - |
| Permethrin* | I | 100 | 931 | Trace |  | 1650 | 1180 |  | 340 | 678 |
| Piperonyl butoxide** | I | 12 | - | - |  | Trace | 25 |  | Trace | - |
| Propargite | I | 2 | 6 | Trace |  | Trace | 3 |  | - | Trace |
| Pyraclostrobin | F | 2 | Trace | - |  | - | - |  | - | - |
| Pyrimethanil | F | 5 | - | Trace |  | Trace | 8 |  | - | Trace |
| Tetramethrin | I | 100 | - | - |  | Trace | - |  | - | - |
| Thymol | V | 2 | 8 | 23 |  | 194 | 30 |  | 20 | 33 |
| Trifloxystrobin | F | 1 | - | - |  | - | Trace |  | 1 | - |

*Was not tested in the 2016-17 study

**MGK-264 and piperonyl butoxide are synergists classified here as insecticides

**Bee bread samples**

S13 Table. Agrochemical residues in bee bread from the August and October 2016 samples. F = fungicide; H = herbicide; I = insecticide; V = varroacide

|  |  |  | August 2016 | |  |  |  |  | October 2016 | | |  |
| --- | --- | --- | --- | --- | --- | --- | --- | --- | --- | --- | --- | --- |
| Agrochemical | Type | LOD | AG1 | AG2 | CA1 | CA2 | MT1 | MT2 |  | AG1  + AG2 | CA1  + CA2 | MT1  + MT2 |
| DMPF | V | 5 | 38 | Trace | 110 | 84 | 45 | 84 |  | 125 | 200 | 125 |
| Acetamiprid | I | 4 | Trace | Trace | - | - | - | - |  | - | - | - |
| Atrazine | H | 4 | Trace | - | - | - | - | - |  | - | - | - |
| Boscalid | F | 10 | - | - | Trace | Trace | - | Trace |  | - | - | - |
| Carbendazim (MBC) | F | 5 | - | - | - | Trace | Trace | - |  | - | Trace | - |
| Chlorothalonil | F | 100 | - | - | - | - | - | - |  | Trace | - | - |
| Chlorpyrifos | I | 5 | 60 | 63 | - | - | - | - |  | - | - | - |
| Coumaphos | V | 3 | - | - | Trace | - | Trace | - |  | Trace | Trace | - |
| Cyhalothrin total | I | 5 | 27 | 8 | - | - | - | - |  | - | - | - |
| DDE p,p’ | I | 5 | Trace | Trace | - | - | - | - |  | Trace | - | - |
| Endosulfan II | I | 10 | - | - | - | - | - | - |  | 13 | - | - |
| Flonicamid | I | 15 | 19 | 42 | - | - | - | - |  | Trace | - | - |
| Fluvalinate | V | 5 | 5 | Trace | 10 | 8 | 15 | Trace |  | Trace | 5 | 5 |
| Hexythiazox | I | 15 | Trace | Trace | - | - | - | - |  | Trace | - | - |
| Imidacloprid | I | 6 | Trace | Trace | - | - | - | - |  | Trace | - | - |
| Indoxacarb | I | 30 | 42 | - | - | - | - | - |  | Trace | - | - |
| Methoxyfenozide | I | 5 | 110 | 350 | Trace | Trace | Trace | Trace |  | 220 | Trace | Trace |
| Piperonyl butoxide** | I | 15 | - | - | Trace | - | Trace | Trace |  | - | Trace | - |
| Propargite | I | 15 | - | - | - | - | - | - |  | - | - | Trace |
| Pyraclostrobin | F | 5 | - | - | Trace | Trace | - | - |  | - | - | - |
| Spinosad | I | 15 | 33 | - | - | - | - | - |  | Trace | - | - |
| Spirodiclofen | I | 5 | - | - | - | 8 | - | - |  | - | - | - |
| Thymol | V | 50 | Trace | Trace | Trace | Trace | - | 100 |  | Trace | Trace | Trace |
| Trifluralin | H | 5 | Trace | Trace | - | - | - | - |  | 5 | - | - |

**Piperonyl butoxide, a synergist, was here classified as an insecticide

**Bee bread samples cont’d**

S14 Table. Agrochemical residues in bee bread in August 2017. F = fungicide; H = herbicide; I = insecticide; V = varroacide

|  |  |  | August 2017 | |  |  |  |  |  |  |
| --- | --- | --- | --- | --- | --- | --- | --- | --- | --- | --- |
| Agrochemical | Type | LOD | AG3 | AG4 |  | CA3 | CA4 |  | MT3 | MT4 |
| DMPF | V | 1.5 | 2 | 252 |  | 257 | 42 |  | 71 | 98 |
| Acetamiprid | I | 2.5 | - | - |  | - | Trace |  | - | - |
| Atrazine | H | 4 | Trace | Trace |  | - | - |  | - | - |
| Azoxystrobin | F | 1 | - | - |  | Trace | - |  | - | - |
| Bensulide* | H | 4 | - | - |  | 6 | - |  | - | - |
| Bifenazate | I | 3 | - | - |  | - | Trace |  | - | - |
| Boscalid | F | 5 | - | - |  | - | Trace |  | - | - |
| Buprofezin | I | 2 | Trace | - |  | - | - |  | - | - |
| Carbendazim | F | 2 | - | - |  | Trace | - |  | - | - |
| Chlorantraniliprole | I | 15 | Trace | Trace |  | - | - |  | - | - |
| Chlorpyrifos | I | 5 | - | 21 |  | - | - |  | - | - |
| Chlorthal-dimethyl (DCPA)* | H | 2 | Trace | Trace |  | Trace | Trace |  | Trace | Trace |
| Cyphenothrin | I | 1000 | - | - |  | - | Trace |  | 2220 | Trace |
| Cyprodinil | F | 2 | - | Trace |  | - | - |  | - | - |
| DDE p,p' | I | 2 | - | Trace |  | - | - |  | - | - |
| DEET* | I | 3 | 6 | 7 |  | - | - |  | 4 | 4 |
| Diuron | H | 1 | - | - |  | 1 | 1 |  | Trace | - |
| Etoxazole | I | 0.5 | - | - |  | 1 | Trace |  | Trace | - |
| Fenamidone | F | 1 | - | Trace |  | - | - |  | - | - |
| Flonicamid | I | 7 | - | 17 |  | - | Trace |  | - | - |
| Flupyradifurone | I | 7 | 135 | Trace |  | - | - |  | - | - |
| Fluvalinate | V | 25 | - | Trace |  | - | - |  | Trace | Trace |
| Hexythiazox | I | 2 | - | 5 |  | - | Trace |  | - | - |
| Methoxyfenozide | I | 1 | 1520 | 89 |  | 3 | 6 |  | 2 | 2 |
| Pendimethalin | H | 20 | Trace | Trace |  | - | - |  | - | - |
| Penthiopyrad* | F | 1 | - | - |  | - | - |  | Trace | - |
| Propamocarb hydrochloride* | F | 2 | - | - |  | 5 | 6 |  | - | - |
| Pyraclostrobin | F | 2 | - | - |  | - | Trace |  | - | - |
| Spirodiclofen | I | 4 | - | - |  | 6 | 8 |  | - | - |
| Tebuconazole | F | 5 | - | - |  |  | 8 |  | - | - |
| Tebufenozide | I | 0.5 | 2 | - |  | - | - |  | - | - |
| Thymol | V | 2 | - | Trace |  | Trace | Trace |  | - | Trace |
| Trifloxystrobin | F | 1 | - | - |  | - | 1 |  | - | - |

*Was not tested in the 2016-17 study

**Bee bread samples cont’d**

S15 Table. Agrochemical residues in bee bread in October 2017. F = fungicide; H = herbicide; I = insecticide; V = varroacide

|  |  |  | October 2017 | |  |  |  |  |  |  |
| --- | --- | --- | --- | --- | --- | --- | --- | --- | --- | --- |
| Agrochemical | Type | LOD | AG3 | AG4 |  | CA3 | CA4 |  | MT3 | MT4 |
| DMPF | V | 1.5 | 104 | 58 |  | 100 | 135 |  | 202 | 152 |
| Azoxystrobin | F | 1 | Trace | Trace |  | - | - |  | - | Trace |
| Bensulide* | H | 4 | - | - |  | - | - |  | - | 37 |
| Boscalid | F | 5 | Trace | Trace |  | - | - |  | - | 17 |
| Chlorantraniliprole | I | 15 | Trace | - |  | Trace | Trace |  | Trace | 18 |
| Chlorothalonil | F | 250 | 819 | 908 |  | - | - |  | 398 | 497 |
| Chlorthal-dimethyl (DCPA)* | H | 2 | 3 | - |  | 3 | 4 |  | 6 | 32 |
| Etoxazole | I | 0.5 | - | 1 |  | - | - |  | - | - |
| Fenpyroximate | I | 3 | Trace | Trace |  | Trace | - |  | - | - |
| Flonicamid | I | 7 | - | - |  | - | - |  | 27 | - |
| Fludioxonil | F | 10 | - | Trace |  | - | - |  | Trace | - |
| Metalaxyl | F | 1 | - | 2 |  | - | - |  | - | 5 |
| Methoxyfenozide | I | 1 | 12 | 11 |  | 7 | 8 |  | 91 | 126 |
| Metolachlor | H | 25 | - | - |  | Trace | - |  | - | - |
| Prodiamine | H | 10 | - | - |  | - | - |  | - | 536 |
| Pronamide | H | 25 | - | - |  | - | - |  | - | Trace |
| Propiconazole | F | 2 | - | 4 |  | - | - |  | - | - |
| Spinosad | I | 7 | - | - |  | - | - |  | - | 33 |
| Spirodiclofen | I | 4 | 42 | 25 |  | - | - |  | - | 6 |
| Thymol | V | 2 | 7 | 5 |  | - | - |  | 13 | 4 |
| Trifluralin | H | 10 | Trace | - |  | - | - |  | - | Trace |

*Was not tested in the 2016-17 study

**Bee bread samples cont’d**

S16 Table. Agrochemical residues in bee bread from the March 2017 samples. F = fungicide; H = herbicide; I = insecticide; V = varroacide

|  |  |  | March 2017 | |  |  |  |  |  |
| --- | --- | --- | --- | --- | --- | --- | --- | --- | --- |
|  |  |  | Almonds | | |  | Blueberries | | |
| Agrochemical | Type | LOD | AG | CA | MT |  | AG | CA | MT |
| DMPF | V | 5 | 50 | 47 | 40 |  | 24 | 69 | 42 |
| Boscalid | F | 10 | - | - | Trace |  | - | - | - |
| Carbendazim | F | 5 | - | Trace | Trace |  | - | - | - |
| Chlorpyrifos | I | 5 | 83 | 31 | 74 |  | - | - | - |
| Coumaphos | V | 3 | Trace | - | - |  | - | Trace | - |
| Cyprodinil | F | 10 | 48 | 31 | Trace |  | Trace | - | Trace |
| DDE p,p’ | I | 5 | Trace | - | Trace |  | - | - | - |
| Difenoconazole | F | 10 | Trace | - | - |  | - | - | - |
| Diflubenzuron | I | 5 | Trace | Trace | Trace |  | - | - | - |
| Emamectin Benzoate | I | 5 | - | - | - |  | Trace | - | Trace |
| Fluometuron | H | 40 | - | - | - |  | Trace | Trace | 80 |
| Fluopyram | F | 5 | Trace | Trace | Trace |  | - | - | - |
| Fluvalinate | V | 5 | Trace | Trace | Trace |  | - | Trace | Trace |
| Methoxyfenozide | I | 5 | Trace | - | - |  | Trace | - | - |
| Pendimethalin | H | 15 | 19 | Trace | 20 |  | - | - | - |
| Pyraclostrobin | F | 5 | Trace | Trace | Trace |  | - | - | - |
| Pyridaben | I | 5 | Trace | Trace | - |  | - | - | - |
| Pyrimethanil | F | 15 | - | - | 1560 |  | - | - | - |
| Pyriproxyfen | I | 5 | 870 | 480 | - |  | - | - | - |
| Thymol | V | 50 | - | - | - |  | Trace | Trace | Trace |

**Bee bread samples cont’d**

S17 Table. Agrochemical residues in bee bread in March 2018. Here “AG” means colonies from the Imperial Valley (Agriculture: Pampas and Pepper), “CA” means from Escondido (Citrus-Avocado: Harrison and Pauma Creek), and “MT” means the higher elevation colonies (Mountain: Charger and Light yards). Clearly hives sent to almonds were being exposed to a far greater spectrum of agrochemicals than those kept in blueberries. F = fungicide; H = herbicide; I = insecticide; V = varroacide

|  |  |  | March 2018 | | |  |  |  |  |
| --- | --- | --- | --- | --- | --- | --- | --- | --- | --- |
|  |  |  | Almonds | | |  | Blueberries | | |
| Agrochemical | Type | LOD | AG | CA | MT |  | AG | CA | MT |
| DMPF | V | 1.5 | 242 | 181 | 156 |  | 402 | 200 | 169 |
| Azoxystrobin | F | 1 | 14 | 13 | 14 |  | - | - | - |
| Boscalid | F | 5 | Trace | 7 | Trace |  | - | - | - |
| Buprofezin | I | 2 | 18 | 16 | 31 |  | - | - | - |
| Chlorantraniliprole | I | 15 | Trace | Trace | Trace |  | - | - | - |
| Chlorothalonil | F | 250 | 1270 | 1320 | 6860 |  | - | 267 | 312 |
| Chlorpyrifos | I | 5 | - | - | 287 |  | - | - | - |
| Chlorthal-dimethyl (DCPA)* | H | 2 | 10 | 11 | 19 |  | 3 | 3 | 3 |
| Cyprodinil | F | 2 | 35 | 34 | 27 |  | - | - | - |
| Diflubenzuron | I | 2 | 17 | 32 | 33 |  | - | - | - |
| Diuron | H | 1 | 4 | 9 | - |  | - | - | - |
| Fludioxonil | F | 10 | - | - | Trace |  | - | - | Trace |
| Fluopyram | F | 1 | 11 | 14 | 12 |  | - | - | - |
| Fluxapyroxad* | F | 2 | 4 | 5 | 6 |  | - | - | - |
| Pendimethalin | H | 20 | 110 | 132 | 118 |  | - | - | - |
| Penthiopyrad* | F | 1 | 101 | 92 | 73 |  | - | - | - |
| Propiconazole | F | 2 | 8 | 8 | 9 |  | - | - | - |
| Pyraclostrobin | F | 2 | 4 | 4 | 5 |  | - | - | - |
| Pyrimethanil | F | 5 | 7 | 6 | 9 |  | - | - | - |
| Pyriproxyfen | I | 1 | 2 | Trace | 3 |  | - | - | - |
| Simazine | H | 20 | Trace | Trace | Trace |  | Trace | - | 22 |
| Spirotetramat | I | 2 | - | - | - |  | - | Trace | - |
| Thymol | V | 2 | - | - | - |  | 10 | 7 | 9 |
| Trifloxystrobin | F | 1 | 2 | 3 | 3 |  | - | - | - |

*Was not tested in the 2016-17 study
